# Resilience of primary healthcare system across low- and middle-income countries during COVID-19 pandemic: a scoping review

**DOI:** 10.1186/s12961-023-01031-4

**Published:** 2023-09-18

**Authors:** Nousheen Akber Pradhan, Amir Ali Barket Ali Samnani, Kiran Abbas, Narjis Rizvi

**Affiliations:** 1 Department of Community Health Sciences, Aga Khan University, Karachi, Pakistan; 2 Present Address: Institute of Health Policy, Management & Evaluation, University of Toronto, Toronto, Canada

**Keywords:** Primary healthcare, Resilience, COVID-19, LMICs

## Abstract

**Introduction:**

Globally, the coronavirus disease 2019 (COVID-19) pandemic tested the resilience of the health system and its shock-absorbing capacity to continue offering healthcare services. The available evidences does not provide comprehensive insight into primary health care (PHC) system functioning across low- and middle- income countries (LMICs) during the pandemic. Therefore, the objective of this scoping review was to generate evidence on the resilience of PHC systems in LMICs during the COVID-19 pandemic.

**Methods:**

A scoping review was carried out utilizing an iterative search strategy using the National Library of Medicine (NLM) and the WHO COVID-19 electronic databases. Data from the identified studies in LMICs were charted in the Preferred Reporting Items for Systematic Reviews and Meta-Analyses extension for Scoping Reviews (PRISMA-ScR) checklist in the first step. The analysis framework was adapted and modified using COVID-19 and health systems resilience framework developed by Sagan et al., Blanchet et al., and the WHO position paper on ‘Building health systems resilience for universal health coverage and health security during the COVID-19 pandemic and beyond’. A total of 26 documents were included on the basis of predefined eligibility criteria for our analysis.

**Results:**

Our review explored data from 44 LMICs that implemented strategies at the PHC level during the COVID-19 pandemic. Most of the LMICs developed national guidelines on sexual, reproductive, maternal, newborn, child, and adolescent health (SRMNCAH). Most of the countries also transformed and reoriented PHC service delivery by introducing digital healthcare services to continue essential services. Task shifting, task sharing, and redeployment of retired staff were some frequently adopted health workforce strategies adopted by most of the countries. Only a few of the countries demonstrated the availability of necessary monetary resources to respond to the pandemic.

**Conclusions:**

The functionality of the PHC system during the COVID-19 pandemic was demonstrated by a variety of resilience strategies across the six building blocks of the health system. To strengthen PHC resilience, we recommend strengthening community-based PHC, cross-sectoral collaboration, establishing surveillance systems, capacity building in financial risk planning, and investing in strengthening the digital healthcare system.

**Supplementary Information:**

The online version contains supplementary material available at 10.1186/s12961-023-01031-4.

## Key Highlights

- A majority of the LMICs adopted resilience strategies related to service delivery and health workforce domains.
- Nigeria was the only country that showed evidence of resilience measures across all six WHO building blocks.
- Only a few countries reported healthcare financing measures.
- Most of the LMICs have developed national guidelines on SRMNCAH to ensure the continuation of healthcare services.
- Interruption in facility-based services shifted the burden to community-based healthcare services and most of the PHC-level services were offered at doorstep or at community sites.
- For essential service delivery, most of the countries had demonstrated transformative capacity that included use of digital platforms (teleconsultation/telemedicine establishing hotline number, WhatsApp messaging), for appointment system, extended timings, etc.
- Health workforce strategies varied from task shifting of health workforce, developing contractual agreements, and extending working hours to redeployment of retired staff in a majority of the countries.

**Supplementary Information:**

The online version contains supplementary material available at 10.1186/s12961-023-01031-4.

## Introduction

Globally, the coronavirus disease 2019 (COVID-19) pandemic tested the resilience of health systems and their shock-absorbing capacity to continue healthcare service provision. In this regard, the role played by primary healthcare (PHC) is crucial, as it is the entry point in the health system. Investing in PHC to make it resilient to the crisis is thus a need that emerged during the COVID-19 pandemic [1]. Health systems resilience is defined as ‘the capacity of health actors, institutions, and populations to prepare for and effectively respond to crises; maintain core functions when a crisis hits; and, informed by lessons learned during the crisis, reorganize if conditions require it’ [2].

Developing and strengthening PHC resilience highlights a range of factors that are addressed in WHO’s 13th General Programme of Work (2019–2023) [3]. This includes ‘increasing and strengthening the capacity of PHC to sustain continuity in essential service delivery in the context of COVID-19 pandemic, moving from fragmentation to reintegration (where routine and emergencies services can be brought under one platform), working multisectoral and intersectoral, ensuring implementation and knowledge exchange and rethinking anti-fragility and resilience’ [3].

The Astana Declaration on PHC in 2018 revitalized the significance of PHC to ensure that everyone from everywhere can enjoy the highest possible attainable standard of health [4]. PHC addresses the broader determinants of health and goes beyond providing healthcare for specific diseases and aims at whole-person care throughout the lifespan. The activities range from health promotion and prevention to treatment for common ailments. The Astana declaration reaffirmed the context of comprehensive PHC and presented three components; (i) primary care and essential public health functions as the core of integrated health services; (ii) empowered people and communities; and (iii) multisectoral policy and action [5].

Under PHC, the focus in many countries remained on the provision of maternal and child healthcare (MCH) services, health education, nutrition, safe water and sanitation, treatment of common diseases and injuries, immunization against infectious diseases, prevention, and control of locally endemic diseases [6]. The continuity of PHC healthcare services was in high demand during the COVID-19 pandemic. Thus, the need for a more resilient PHC infrastructure was recognized. As the COVID-19 crisis took a toll on health systems around the world in 2020, the entry point in the healthcare delivery system was shifted from PHC to hospitals [6]. This created intense pressure on already strained hospitals caring for sick patients, and thus the role of PHC got overlooked.

As the COVID-19 pandemic swept the world, the role and function of PHC varied greatly across the world. A lot of LMICs managed to continue the delivery of healthcare services during the COVID-19 pandemic with multiple challenges. For instance, India reported infrastructural issues in PHC with limited physical capacity [7]. The operation of outpatient services, specifically related to MCH, faced significant disruptions (*P* < 0.001) during the COVID-19 pandemic [7]. Despite good PHC infrastructure in Bangladesh, challenges were observed in the provision of quality PHC services during the initial phase of the COVID-19 pandemic [8]. Attendance in outpatient and inpatient units reduced substantially, alongside the proportion of fully immunized children dropping by 50% [8]. Likewise, the PHC system in Iran was also affected and led to reduced service utilization by midwives, physicians, dentists and mental health experts [9]. The reported challenges include insufficient knowledge of effective virus management and control strategies, inadequate use of strong PHC capacities, and potential to manage and control the COVID-19 crisis, resulting in reduced service utilization [9]. Like other countries, diagnostic tests for severe acute respiratory syndrome coronavirus 2 (SARS-CoV-2) were widely distributed in Brazil [10–13]. The issues were reported with the accessibility of diagnostic tests across varied population groups, alongside delays in testing results and reporting [14].

Pakistan demonstrated discontinuity in essential healthcare services during the early phase of the COVID-19 pandemic with the closure of PHC centres [15], with millions of children in Pakistan missing out on polio vaccination during the earlier phase of the pandemic [9]. The lockdown imposed by the COVID-19 pandemic led to serious disruptions in routine healthcare services at the outreach and facility levels [15]. At the grassroots level, door-to-door services by lady health workers (LHWs) were also affected due to the fear of contagion [15].

Some good examples of PHC reorientation emerged from high-income countries to respond to COVID-19, such as Europe reporting three models of PHC [16]: (i) ‘Multi-disciplinary primary care teams for the emergency response. The activities included facility-based testing and triage, telephone-based triage, COVID-19 case tracing, home-based monitoring, and delivering a vaccine against COVID-19; (ii) PHC providers prioritizing vulnerable patients, this was inclusive of providing services to vulnerable people such as the provision of medicine to anyone with a prescription during lockdowns, minimum durations of prescriptions were introduced, etc., and (iii) Digital solutions to enhance the effectiveness of the PHC response to both COVID-19- and non-COVID-19-related care [16]. Activities included remote consultation, remote sick leave, electronic prescriptions, to mention a few.

Global evidence of the PHC system’s functioning during the COVID-19 pandemic is continuing to evolve. Therefore, a need for collective evidence on how PHC demonstrated resilience during the COVID-19 pandemic in LMICs was addressed by synthesizing and documenting pieces of evidence on the resilience of PHC systems among resource-constrained countries. The overall objective of this scoping review was therefore to generate and synthesize evidence from LMICs on the resilience of PHC systems during the COVID-19 pandemic.

## Methods

### Study design

To synthesize evidence from LMICs on the resilience of PHC systems during the COVID-19 pandemic, a scoping review was carried out. A comprehensive review of the literature on the PHC system’s shock-absorbing capacity across LMICs is needed to understand how PHC operated during the COVID-19 pandemic. Therefore, a scoping review was deemed a reasonable review for this research. The study protocol was registered with Figshare on 22–12-2022 (https://figshare.com/articles/preprint/Scoping_Review_protocol_V3_final_docx/21763673). The scoping review approach proposed by Arksey and O’Malley [17] was used as below.

### Step 1: establish a scoping review question(s)

What is the available evidence on the resilience of PHC systems during the COVID-19 pandemic in LMICs?

### Step 2: identify relevant studies

The published literature was sought using the online search engines of the National Library of Medicine (NLM) and WHO COVID-19 research databases. The search terms were applied and adapted as appropriate to the syntax of each database. Key research terms composed of those obtained via subject headings of databases, for example, medical subject headings (MeSH). For the grey literature search, the WHO Institutional Repository for Information Sharing (IRIS), and Google Scholar were considered.

To map out the existing evidence related to the resilience of PHC in LMICs, a concentrated search strategy was used (Table 1). The search strategy was carried out using search terms related to the ‘concepts of interest’ (resilience-enhancing strategy at the PHC level), ‘context’ (COVID-19 pandemic), and ‘population’ (LMICs). The search strategies for the selected peer-reviewed database remained similar; however, the concise search term was applied for the grey literature search to minimize duplication with electronic data search.

**Table 1:** Search terms by database

Source	Type of literature	Search terms
National Library of Medicine (NLM) and WHO COVID-19 research databases	Peer-reviewed	(“resilience”[all fields] OR “resilient”[all fields], OR “health service resilience” [all fields” OR “capacity”[all fields] OR “adaptation”[all fields] OR “strength”[all fields] OR “Strengthening” [all fields] OR “responsiveness”[all fields] OR “preparedness”[all fields] OR “resourcefulness” [all fields] OR “recovery”[all fields] AND (primary health care OR PHC OR medical care OR health services OR health care systems) OR “service delivery”[tiab] OR “health workforce”[tiab] OR “health information systems”[tiab] OR “access to essential medicines”[tiab] OR “financing”[tiab] OR “leadership”[tiab] OR “governance”[tiab]) AND (“COVID-19”[tiab] OR “Coronavirus”[tiab] OR “SARS-CoV-2″[tiab] OR “COVID pandemic”[tiab] OR “Corona”[tiab]) AND (“low middle income countries”[all fields])
WHO Institutional Repository for Information Sharing (IRIS) and Google Scholar	Grey literature	Resilience AND Primary health care AND COVID-19

### Step 3: study selection and screening

The scoping review was conducted following the Joanna Briggs Institute (JBI) methodology [18]. The JBI population, concept, and context (PCC) mnemonic was used to develop the title, question, and inclusion and exclusion criteria. The PCC framework facilitated identifying the main concepts embedded in the research question and also informed the search strategy. The detailed inclusion and exclusion criteria are outlined in Table 2.

**Table 2:** Inclusion and exclusion criteria for the scoping review

Theme	Inclusion	Exclusion
Population (countries)	The population includes countries belonging to LMICs as defined by the World Bank, including Afghanistan, Albania, Algeria, American Samoa, Angola, Argentina, Armenia, Azerbaijan, Bangladesh, Belarus, Belize, Benin, Bhutan, Bolivia, Bosnia and Herzegovina, Botswana, Brazil, Bulgaria, Burkina Faso, Burundi, Cabo Verde, Cambodia, Cameroon, Central African Republic, Chad, China, Colombia, Comoros, Democratic Republic of the Congo, Republic of the Congo, Costa Rica, Côte d’Ivoire, Cuba, Djibouti, Dominica, Dominican Republic, Ecuador, Egypt, El Salvador, Equatorial Guinea, Eritrea, Eswatini, Ethiopia, Fiji, Gabon, Gambia, Georgia, Ghana, Grenada, Guatemala, Guinea, Guinea-Bissau, Guyana, Haiti, Honduras, India, Indonesia, Iran, Iraq, Jamaica, Jordan, Kazakhstan, Kenya, Kiribati, Democratic People’s Republic of Korea, Kosovo, Kyrgyz Republic, Loa PDR, Lebanon, Lesotho, Liberia, Libya, Madagascar, Malawi, Malaysia, Maldives, Mali, Marshall Island, Mauritania, Mauritius, Mexico, Micronesia, Moldova, Mongolia, Montenegro, Morocco, Mozambique, Myanmar, Namibia, Nepal, Nicaragua, Niger, Nigeria, North Macedonia, Pakistan, Palau, Papua New Guinea, Paraguay, Peru, the Philippines, Russian Federation, Rwanda, Samoa, São Tomé and Príncipe, Senegal, Serbia, Sierra Leone, Solomon Islands, Somalia, South Africa, South Sudan, Sri Lanka, St. Lucia, St. Vincent and the Grenadines, Sudan, Suriname, Syrian Arab Republic, Tajikistan, Tanzania, Thailand, Timor-Leste, Togo, Tonga, Tunisia, Turkiye, Turkmenistan, Tuvalu, Uganda, Ukraine, Uzbekistan, Vanuatu, Vietnam, West Bank and Gaza, Yemen, Zambia and Zimbabwe.	Papers and reports from high-income countries (HICs) or upper-middle-income countries (UMICs) were not considered (published and grey literature).
Concept	All the articles, reports or policy guidance documents that have mentioned any aspect of resilience, innovations, modification and adaptations in protocol and service delivery during the COVID-19 pandemic in offering PHC services at the facility or community levels, utilizing any WHO building blocks from the framework to document the strategies/interventions adopted study recommendations, were considered for this review.	Any document or article that has no connection with any aspect of PHC were not considered. Furthermore, if the document/s did not touch upon any aspect of resilience strategies or any of the components of the WHO building blocks, it was excluded.
Context	Resilience-enhancing strategies must be considered, keeping the COVID-19 pandemic context at the level of PHC.	Any resilience-enhancing strategies meant for a reason other than the COVID-19 pandemic (such as drought, flood, other communicable diseases, conflicted law and order situations, etc.) were not considered.
Additional filters		
Article type	Original research, reports, commentary or editorial, systematic, scoping or rapid review, letter, policy document, policy brief, advisories, strategy document, action plan, and meeting document.	Media news, clinical trials, case studies, case reports and case series.
Reporting/language	English language	Other languages
Date of publication	Eligible papers or reports published from 1 January 2020 until 31 December 2022 were considered for this review.	Papers submitted before the COVID-19 pandemic (2019) were not considered.

**Fig. 1:**
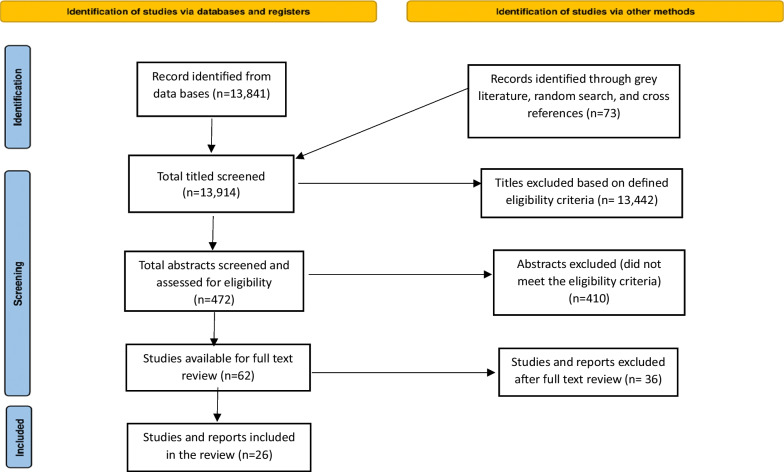
Consort flow diagram illustrating the process of data screening and selection

Article screening and selection were managed by the two researchers experienced in scoping review and health systems. The initial screening of the paper was carried out by reviewing the title first. If the title was considered appropriate, the second step was to review the abstract. If the abstract met the inclusion criteria, this was followed by reviewing the entire article and/or report. Any identified discrepancies between the two researchers were resolved through discussion and involving the principal investigator (PI). We identified a total of 13,841 articles and reports from the NLM and WHO COVID-19 databases. Furthermore, a total of 73 records were identified through a grey literature search. Post-screening, 13, 442 articles were excluded as they did not fulfil the eligibility criteria. A total of 472 abstracts were assessed for screening. Out of these, 410 abstracts were classified ineligible. Therefore, a total of 62 papers and reports were included for full text review. Of these, 28 articles and reports were selected for analysis. Two papers were later dropped due to lack of consensus among the reviewers. Thus a total of 26 articles and reports were included in the final analysis (Fig. 1). The inter-reviewer agreement rate was 93%.

### Step 4: data charting and analysis

The Excel-based data extraction sheet comprised of 12 items including literature code, publication year, source and citation, title of the document, country, publication type, component of WHO building block/s addressed, targeted service, health setting(s), strategies adopted by the LMICs, main outcome/impact, and key recommendations/lessons learned (Table 3). One reviewer charted the relevant data and insights from the included studies using the developed instrument that served as our guideline, while the second reviewer ensured that the charting process was consistently applied. Once data was entered and finalized, the original data extraction sheet (Additional file 1) was transferred to a Word file (Table 7).

**Table 3:** Data charting categories and definitions

Categories	Definitions
Code	Specific identifiable document code
Publication date	Month and year of publication
Source citation	Publication bibliographical information
Title	Study/literature title
Country	Name of the country where the study took place or the focus of the publication
Publication type	Type of document included for the study as outlined in the eligibility criteria (journal article, any report, policy guidance, guidance notes, document, editorial, rapid review, policy brief, etc.)
Components of WHO building blocks	WHO six building blocks include leadership and governance, health workforce, health financing, service delivery, health information system, and supplies and equipment
Targeted service	Essential PHC services include immunization, nutrition, child health, maternal health, adolescent health, mental health, elderly care, reproductive health, health promotion, health education surveillance, monitoring, community engagement, etc.
Health settings	Health settings include community level, PHC facility level, or policy/system level (with a focus on PHC)
Strategies adopted by countries	Resilience measures (absorptive, adaptive, and transformative) taken by the country or any other action whose principal intent is towards continuity of PHC services or reducing the burden of the COVID-19 pandemic on primary care/essential services
Outcome	The outcome is in line with the objective of the paper. Concerning the scoping review, this includes what measures were adopted during the pandemic
Recommendations	Considering coping measures and overall response as well as what suggestions and recommendations were made or suggested in the included literature

### Analysis framework

Existing literature yields multiple approaches to assessing health systems’ resilience [1, 19–21]. Hence, to obtain a holistic perspective on PHC system resilience across LMICs, a health systems approach was the best fit for our research question. The analysis framework for the scoping review was adapted and modified from the framework of COVID-19 and health systems resilience developed by Sagan et al. [22], Blanchet et al. [20], and the WHO position paper on ‘Building health systems resilience for universal health coverage and health security during the COVID-19 pandemic and beyond’ [21]. Our framework includes all six health systems’ building blocks (governance, service delivery, health workforce, financing, information system, and supplies and equipment). Under each building block, a set of strategies is included. A total of 14 strategies were included. To operationally define these strategies, a description of the strategies was also added to bring transparency to our analysis in extracting the relevant information (Table 4).

**Table 4:** Exploring resilience in PHC system using WHO building blocks framework

Strategies	Elements
Governance	
Adequate and effective leadership	1. Having a clear vision for an effective PHC strategy 2. Reliance on the best available evidence but adopting the precautionary principle where evidence is uncertain 3. Culture of learning; ability to act fast; effective and transparent communication (especially regarding uncertainty); 4. Community participation; participation in the international community (e.g. joint procurement, clinical networks, etc.)
Coordination of activities across government departments and among key stakeholders	5. Presence of a clear and widely understood strategy 6. Coordination within government (horizontal and vertical) and key stakeholders including civil society
Financing	
Ensure sufficient monetary resources in the system and flexibility to reallocate and inject extra funds into the system	7. Ensuring sufficient monetary resources in the system and flexibility to reallocate and inject extra funds into the system 8. Ensuring the financing arrangement prioritizes the provision of essential PHC services 9. Any evidence of a disaster risk financing mechanism
Purchasing flexibility and reallocation of funding within the system to meet changing needs	10. Ability to quickly adapt procurement and payment systems while maintaining transparency, timeliness, and quality, including measures to prevent corruption
Health workforce	
Appropriate level and distribution of human resources	11. Ensuring a sufficient supply of personal protective equipment for the workforce 12. Workforce available and well deployed across urban and rural areas to provide services
Recruitment and capacity building of the health workforce	13. Ability to mobilize additional human resources via training of existing workforce or adapting their roles, alongside recruiting and training volunteers
Motivated and well-supported workforce	14. Ensuring mental health (e.g. psychological counselling), family (e.g. childcare), physical (e.g. respite breaks), and financial support for healthcare workers
Service delivery	
Absorptive, adoptive and transformative capacity	15. Absorptive capacity: PHC continues to deliver the same level (quantity, quality and equity) of basic healthcare services and protection to populations 16. Adoptive capacity: determines the health system actors to adapt to the crisis to deliver the same level of healthcare services with fewer and/or different resources 17. Transformative capacity: the ability of health system actors to transform and reorient the functions and structure of the health system to respond to a changing environment
An essential package of services	18. Having a package of services that is properly resourced, organized and distributed 19. Identifying vulnerable population groups (ensuring that appropriate data are collected) and ensuring adequate access to services 20. Evidence of continuing essential public health functions
Evidence of services related to COVID-19 pandemic	21. Having strong (or strengthening) public health capacity (with a system to find, test, trace, isolate, and support
Ability to deliver services safely	22. Having strong (or strengthening) primary health in maintaining non-COVID essential services to catchment populations 23. Mechanisms in place to ensure effective implementation of infection prevention and control in healthcare settings
Information system	
Surveillance enabling the timely detection of data during COVID-19 pandemic	24. Evidence of active and/or passive surveillance system 25. Evidence of timely data generation
Effective communication systems and flows	26. Having (or establishing) well-functioning communication channels linked to lines of accountability, including hard and soft infrastructure
Supplies and equipment	
Personal protective equipment (PPE) and other essential supplies and equipment for PHC service delivery	27. Stock of PPE 28. Adequate stock of essential equipment and supplies

The framework facilitated the exploration of resilience-enhancing strategies at the PHC level that were adopted by the LMICs during the COVID-19 pandemic. The analysis was carried out at two levels. Firstly, the findings were categorized using the WHO building blocks. Later, the findings were sorted into ‘strategies and elements’ across the building blocks, as guided by our framework (Table 4). To overcome the researcher’s subjectivity in categorizing the findings, the second reviewer reviewed all the findings to validate the assortment, and a consensus was achieved in the team by engaging the third reviewer (Principal Investigator). Findings that were beyond the scope of PHC were excluded, and those that portray a health systems level with a strong possibility of supporting PHC were kept under the respective elements.

### Step 5: synthesizing and reporting of findings

The results were synthesized descriptively in the tabulated format using an Excel spreadsheet. The results were described in the narrative form by relevant themes (Additional file 2), as highlighted in the analysis framework. This includes a detailed description and charting of the relevant data in specified categories for each of the six building blocks, as depicted in the analysis framework. Furthermore, the results were also tabulated and synthesized at the country level by selecting the key findings from the analysis using the WHO building blocks framework (Table 5).

**Table 5:** Examples of key findings at the country level (refer Additional file 4)

[SR number]	Countries		Governance	Financing	Workforce	Service delivery	Information system	Supplies and equipment
1	**Albania**		NIL	NIL	**Motivated and well-supported workforce** Monthly salary raises for the entire duration of the crisis from the central government [SR-4]	**Evidence of service related to COVID-19** PHC providers supported public health surveillance teams in contact tracing [SR-4]	NIL	NIL
2	**Bangladesh**		**Adequate and effective leadership** National guidelines on MNCH&FP Services had been developed in the context of COVID-19 [SR-13] **Coordination of activities across government departments and among key stakeholders** Multisectoral coordination committee formed at the facility level [SR-25]	NIL	**Recruitment of health workforce** Recruitment of additional doctors, nurses and midwives [SR 12] to ensure the continuity of essential services; 2000 medical doctors and 5000 nurses were newly recruited and posted to primary-level health facilities [SR-25] **Capacity building of the health workforce** Trained Rohingya refugees (worked as CHVs) ensured that routine healthcare support, including immunizations, management of non-communicable diseases, and maternal and child health continued in the community [SR-07] Virtual orientation on maternal, newborn, and child health guidelines was provided to healthcare workers. In addition, virtual platform for training was offered to staff on revised integrated management of childhood illnesses (IMCI) protocols [SR-12] **Motivated and well-supported workforce** Provision of accommodation close to the facility and transport to the facility [SR-25]	**Adoptive/ transformative capacity** 24/7 Telecounselling services were offered via government call centres [SR-12] Digital platforms (WhatsApp, Viber), hotlines and telemedicine and support services on maternal, child, reproductive, and adolescent health provided (some 24/7) [SR-12] **Evidence of continuing essential public health function** Community and volunteer groups were engaged to help distribute healthcare information to families [SR-12] **Evidence of service related to COVID-19** A dedicated cadre of health workers introduced for COVID-19 treatment [SR-13]	**Surveillance and timely generation of data during the COVID-19 pandemic** Early and continuous monitoring of data at the PHC level was fundamental to informing the response strategies to restore the availability and use of essential health services [SR-11] **Effective communication systems and flow of information** Frequent online meetings with PHC facilities to support the maintenance of essential health services [SR-25] Scaled-up regular monitoring of essential health service activities at primary level, that is, community clinics [SR-25]	**Personal protective equipment (PPE) and other essential supplies and equipment for PHC service delivery** PPE for health workers, and infection prevention & control (IPC) in all health facilities ensured [SR-12]
3	**Bhutan**		**Adequate and effective leadership** Operational guidelines for the continuity of SRMNCAH were created at the health systems level, and guidelines/standard operating procedures (SOPs) were prepared to facilitate the implementation of contingency plans and policies at the facility level [SR-13] The efficient functioning of health systems was boosted by the implementation of stewardship and good governance [SR-13]	**Ensuring sufficient monetary resources in the system and flexibility to reallocate and inject extra funds into the system** Financial support from the private sector to ensure government mobilization of PPEs was mobilized to supplement existing stock [SR-12]	**Capacity building of health workers** Continued medical education and online capacity development of healthcare providers [SR-13] **Appropriate level and distribution of human resources** Allocation of human resources for health [SR-13]	**Absorptive capacity** Contingency plans were developed to ensure the provision of essential healthcare services and micro-plans for health facilities were activated [SR-13] **Ability to deliver services safely** Prevention of infections among health workers was addressed through the operational guidelines of MCH services [SR-13]	**Effective communication systems and flow of information** Social mobilization and community empowerment activities were undertaken by the government, including awareness-generation campaigns on essential healthcare services and emergency services during the lockdown [SR-13]	NIL
4	**Bolivia**		NIL	NIL	**Capacity building of the health workforce** Use of digital technology for the orientation and training of health personnel [SR 12]	**Transformative capacity** Strengthened referral systems to higher-level of public and private centres for the management of complications related to maternal and newborn health and offered abortion services [SR-12] Provided virtual care through telemedicine and WhatsApp [SR-12] Use of digital technology for educating the public [SR-12] Government-supported youth initiative programs that utilized WhatsApp and call centres to provide health information and service referrals [SR-12] **Absorptive capacity** National Network of People Living with HIV and AIDS and National Sexually Transmitted Infection/HIV/AIDS Programme provided RT for people living with HIV/AIDS [SR12] **Ability to deliver services safely** Scheduling of prenatal and postnatal visits to reduce the number of women in close contact at health facilities [SR-12] **Evidence of service related to COVID-19** Screening, triage, and isolation for COVID-19 patients [SR-12]	**Surveillance enabling the timely detection of shocks and their impact** RedBol12 (National Network of People Living with HIV and AIDS) and National Sexually Transmitted Infection/HIV/AIDS Programme coordinated to support surveillance, information, and referral centres Monitoring of population health problems, and health education for the general population [SR-12]	**Personal protective equipment (PPE) and other essential supplies and equipment for PHC service delivery** PPE for health workers, and IPC in all health facilities [SR-12]
5	**Bosnia and Herzegovina**	**Adequate and effective leadership** Developing formal channels for transferring science into policy by newly establishing expert advisory groups [SR-4]		NIL	**Motivated and well-supported workforce** Financial compensation to support the health workforce in the form of one-time bonus payments [SR-04]	NIL	NIL	NIL
6	**Botswana**		NIL	NIL	NIL	**Absorptive capacity** Extension of review periods for stable patients with a history of NCD and medication refills at pharmacies without prior doctor consultation [SR-24] Community health workers (CHWs) provided home visits for follow-up of patients with HIV, tuberculosis (TB), mental disorders, postnatal care, and palliative care needs [SR-24] **Ability to deliver services safely** Delivered medications, non-pharmaceutical equipment, and supplies to people at home living with chronic conditions, thus protecting them from potential exposure at primary care facilities and in public transport [SR-24] **An essential package of services** Public health education campaigns were carried out through community organizations and social media [SR-24]	NIL	NIL
7	**Brazil**		NIL	NIL	**Appropriate level and distribution of health workforce** Relocation of specialists to primary health centres to expand access to care for pregnant women [SR-12] **Recruitment of health workers** Emergency recruitment of extra health workers [SR-12]	**Absorptive capacity** Vaccination teams dispatched to speciality centres and basic health units in urban and rural areas for immunization Mobile units were organized for vaccination campaigns (immunization) [SR-12] Scheduling of antenatal care (ANC) appointments farther apart, except for the final stage of pregnancy [SR-12] Prioritization of high-risk prenatal care at obstetric clinics, with low-risk prenatal care assigned to basic health units [SR-12] **Adoptive/transformative capacity** Contracting arrangements were made with universities to provide obstetric services, such as ultrasounds and echocardiograms, for pregnant women Paediatric services were established within emergency care unit with semi-intensive care beds for patients with symptomatic COVID-19 [SR-12] Implementation of telemedicine consultations to increase user access and reduce in-person contact at health facilities [SR-12] **An essential package of services** Implementation of Elderly Health Home Record Book to record personal, social and family data; health conditions; health behaviours and vulnerabilities; and guidance on self-care [SR-12]	NIL	**Personal protective equipment (PPE) and other essential supplies and equipment for PHC service delivery** PPE for health workers, and IPC in all health facilities [SR-12]
8	**Bulgaria**		NIL	NIL	**Motivated and well-supported workforce** Developed helplines or apps and online support for the mental health of health workers [SR-4]	NIL	NIL	NIL
9	**Cameroon**		NIL	NIL	NIL	**Absorptive capacity** Special and catch-up campaigns in certain areas for cholera, polio, and measles vaccines immunization [SR-12] **Adoptive/transformative capacity** Modification of frequency of vaccination clinics and outreach schedules from weekly to monthly immunization and increased intervals between clinic visits (MNCAH) [SR-12] Expansion of role of CHW to include family planning (FP) and vaccination counselling and to assist with adherence to public health programs [SR-12] Telehealth appointments for ANC and PNC [SR-12] **Evidence of service related to COVID-19** Screening, triage and isolation for patients with COVID-19 [SR-12] **Ability to deliver services safely** Community-based ANC for pregnant patients with positive for asymptomatic/mild COVID-19 [SR-12]	**Effective communication and flow of information** Broadcast, print and social media campaigns to raise awareness of the reopening of health facilities for routine services [SR-12]	**Arrangements for the continuity of medicines, supplies and equipment** National carrier arrangement to ensure uninterrupted medicine supply chain [SR-12]
10	**Congo**		NIL	NIL	**Appropriate level and distribution of health workforce** Redeployment and accelerated retraining of nursing staff as midwives for uncomplicated births [SR-12] **Capacity building of health workers** Online training modules for obstetrics, neonatal emergency care and other RMNCAH services [SR-12]	**Adoptive/transformative capacity** Teleconsultation services provided for pregnant women who were unable to travel to health facilities. Services included ANC and prescriptions [SR-20] Mobile and on-site community vaccination campaigns were organized [SR-12]	**Effective communication and flow of information** Media channels used in the community and in hospitals to raise awareness of breastfeeding [SR-12]	**Personal protective equipment (PPE) and other essential supplies and equipment for PHC service delivery** PPE for health workers, and IPC in all health facilities [SR-12]
11	**Ethiopia**		**Coordination of activities across government and key stakeholders** Ethiopian Public Health Institute (EPHI) launched the COVID-19 Plan Revitalization Movement, which focussed on enhancing the COVID-19 response by increasing community awareness through risk communication and community engagement, detection capacity, and improving the quality of care at national and subnational levels [SR-14] **Adequate and effective leadership** Strengthening of public health at sub-national levels: engagement of communities in communications and testing campaigns [SR-16]	NIL	**Capacity building of health workers** Mobile learning platform (LEAP), interactive text messages, and interactive voice response for training Health Education Workers (HEWs) on RMNCAH services during COVID-19 (Ethiopia) [SR-12]	**Adoptive/transformative capacity** Reduced frequency of follow-ups for children with malnutrition and increased ready-to-use food rations Reduced the frequency of prevention of mother-to-child transmission of HIV (PMTCT) appointments to every 3 months (a model known as 3MMD) [SR-12] **Ability to deliver services safely** Implementation of prevention measures and respiratory hygiene measures, such as face coverings, physical contact [SR-14] **Evidence of service related to COVID-19** An establishment of new public health laboratories and linkages with private (including research) laboratories [SR-16]	**Surveillance enabling the timely detection of shocks and their impact** Ethiopia upgraded the surveillance system from manual to DHIS 2, which is a sophisticated, open-source, and customizable software platform that supports data collection, analysis and visualization [SR-16] **Effective communication and flow of information** Public health broadcasts on protecting mental health during lockdown [SR-12]	**Arrangements for the continuity of medicines, supplies and equipment** Reinforced support for monitoring and procurement of RMNCAH commodities [SR-12]
12	**Ghana**		NIL	NIL	NIL	**Adoptive/transformative capacity** Telephone consultations, telemedicine and web-based platforms were used to keep patients from attending health facilities [SR-24] **Ability to deliver services safely** Delivered medications, non-pharmaceutical equipment and supplies to people at home living with chronic conditions, thus protecting them from potential exposure at primary care facilities and in public transport [SR-24] **Evidence of service related to COVID-19** Home visits and contact tracing at the district, sub-district and community levels [SR-22] **An essential package of services** Public health education campaigns were carried out through community organizations and social media [SR-24]	**Effective communication and flow of information** Risk communication activities for maternal healthcare were established (these cover community engagement and the development of broadcast materials) [SR-22] **Surveillance enabling the timely detection of shocks and their impact** Surveillance system in placed [SR-22]	NIL
13	**Haiti**		NIL	NIL	NIL	**Adoptive/transformative capacity** Adoption of national guidelines for safe maternal healthcare [SR-23] **Absorptive capacity** Preservation of CHW/village health worker (VHW) activities related to maternal health [SR-23]	NIL	NIL
14	**India**		**Adequate and effective leadership** Two guidelines were developed: (1) Enabling delivery of essential health services during the COVID-19 outbreak (14 April 2020) and (2) Guidelines on the provision of RMNCAH + N services during and post-COVID-19 pandemic (24 May 2020) [SR-13]	**Ensuring sufficient monetary resources in the system and flexibility to reallocate** Insurance cover for healthcare workers was offered and payments to families were also announced in the unfortunate event of the death of a healthcare worker in the line of duty [SR-13]	**Appropriate level and distribution of health workforce** Additional nursing staff redeployed from other facilities [SR 12] **Capacity building of health workers** CHWs were trained in COVID-19 prevention activities including preventing the spread of rumours and misinformation in the community [SR-7] Department of Personnel and Training launched a learning platform (https://www.pradhanmantriyojana.co.in/igot-onlinetraining-portal-covid/) for all frontline workers for training and to provide updates on coping with the pandemic [SR-12] Telemedicine training and webinar series on providing safe RMNCAH care during COVID-19 [SR-12] **Recruitment of health workforce** Recruitment of contractual staff and re-recruitment of retired health staff [SR-8]	**Adoptive/transformative capacity** Mapped all health facilities at block, district and state levels and reorganized healthcare service delivery for COVID-19 and non-COVID-19 care [SR-10] Telemedicine digital platform (E-Sanjeevani) was offered for caregivers, medical professionals and those seeking health services (India) [SR-12] WONDER app: dedicated mobile-based application developed in Darbhanga, Bihar for tracking high-risk pregnancies and providing referrals and ambulance service [SR-12] **An essential package of services** The promotion of the ‘new normal’ behaviours have been integrated into national CHW guidelines; CHWs have played a critical role in promoting these ‘new normal’ behaviours by engaging with their communities to understand people’s perceptions and by framing the behaviours in the relevant context [SR-7] **Evidence of service related to COVID-19** Standard operation procedures for screening, triage, and isolation for suspected maternal and newborn COVID-19 cases were developed [SR-12] **Ability to deliver services safely** Private doctors involved in emergency ANC services, ANC services strengthened by home visits; continuity of services was maintained using door-to-door distribution of medicines for NCD, TB, and ANC [SR-8]	**Surveillance enabling the timely detection of shocks and their impact** Effective pandemic management was carried out through an integrated disease surveillance system [SR-10]	**Personal protective equipment (PPE) and other essential supplies and equipment for PHC service delivery** PPE for health workers, and IPC in all health facilities [SR-12]
15	**Indonesia**		NIL	NIL	NIL	**Adoptive/transformative capacity** IMCI and COVID-19 protocols were combined with triage protocols (Indonesia) [13] Pregnancy and childbirth services were modified, in addition to family planning services and methods [13] Introduce digital technology to fight against COVID-19 infodemics [SR-13] **Evidence of service related to COVID-19** CHWs have been providing COVID-19-related information to the community [SR-7] **Ability to deliver services safely** Services were scheduled on the basis of appointments and implemented with IPC norms [SR-13]	NIL	NIL
16	**Kenya**		**Adequate and effective leadership** The government issued national-level policy guidance related to RMNCH service continuity [SR-20] **Coordination of activities across government and key stakeholders** The government of Kenya established the National Emergency Response Committee (NERC) on coronavirus to ensure cohesive and effective coordination of the country’s preparedness efforts, mandate included coordinating disease surveillance, building the capacity of healthcare workers, coordinating the supply of tests and other medical supplies, and developing mitigation strategies [SR-14]	NIL	**Appropriate level and distribution of health workforce** Staff were redeployed within and between facilities [SR-1] **Motivated and well-supported workforce** Staff received counselling and psychosocial support on how to cope with the pandemic [SR-1]	**Absorptive capacity** Special guidelines were put in placed to ensure that pregnant and breastfeeding women continued to receive the requisite care without any disruption, guidelines also ensured that no pregnant woman was refused MNCH services due to barriers such as cost [SR-1] **Adoptive/transformative capacity** Reorganization of space for service provision: screening and triage area. Sitting positions and waiting areas were established for clients coming to the facility were marked [SR-1] Phone-based guidance for providing birthing and PNC counselling services [SR-20] **Ability to deliver services safely** Guidelines on the provision of MNCH services during the COVID-19 pandemic, which emphasized necessary preventive measures to be put in place when offering services in the context of the COVID-19 pandemic [SR-1] **An essential package of services** Utilization of community services during COVID-19: health education and sensitization of the community [SR-1] **Evidence of service related to COVID-19** Increased diagnostic capacity for COVID-19 [SR-14]	**Effective communication and flow of information** Communication of COVID-19-specific policy/mitigation measures and messaging instituted [SR-01]	**Ensured a sufficient supply of PPE for the workforce** Provided PPE to all county health facilities [SR-14]
17	**Kyrgyzstan**		NIL	**Ensuring sufficient monetary resources in the system and flexibility to reallocate** Complemented government funds with humanitarian aid [SR-04]	**Motivated and well-supported workforce** Financial compensation to support the health workforce in the form of one-time bonus payments [SR-04]	NIL	NIL	NIL
18	**Lesotho**		NIL	NIL	NIL	**Adoptive/transformative capacity** Adoption of national guidelines for safe maternal care {SR-23] **Absorptive capacity** Preservation of activities of CHWs/VHWs related to maternal health [SR-23]	NIL	NIL
19	**Liberia**		NIL	NIL	NIL	**Adoptive/ transformative capacity** Adoption of national guidelines for safe maternal care [SR-23] **Absorptive capacity** Preservation of activities of CHWs/VHWs related to maternal health [SR-23] **An essential package of services (including health education and awareness)** Implementation of communication campaigns [SR-23]	NIL	**Adequate stock of essential equipment and supplies** Supply chain strengthening [SR-23]
20	**Malawi**		NIL	NIL	**Recruitment of health workers** Recruitment of new health professionals [SR-23] **Appropriate level and distribution of human resources** Increased the workload of community health workers, known in the Malawi health system as health surveillance assistants, responsible for surveillance activity [SR-3] CHWs tasked with additional responsibilities, including sharing information about COVID-19 and prevention measures, making home visits to people who tested positive for COVID-19, and disinfecting their homes as they recovered [SR-3]	**Adoptive/transformative capacity** Adoption of national guidelines for safe maternal care [SR-23] Reorganization of space for service provision to reduce congestion, only pregnant women were admitted, and no companions were allowed to stay [SR-3] **Absorptive capacity** Preservation of activities of CHWs/VHWs related to maternal health [SR-23] **An essential package of services** Implementation of communication campaigns [SR-23] **Ability to deliver services safely** Service providers were required to always have facemasks on and to observe social distancing [SR-3]	**Surveillance enabling the timely detection of shocks and their impact** CHWs assisted in surveillance activities [SR-3] **Effective communication and flow of information** Communication of COVID-19-specific policy/mitigation measures and messaging instituted [SR-03]	NIL
21	**Maldives**		NIL	NIL	**Capacity building of health workers** Online refresher training was offered to health professionals in relevant SRMNCAH areas [13]	**Adoptive/transformative capacity** Outreach programs were conducted for ensuring timely ultrasound scans and ANC consultations for clients [SR-13] **Evidence of service related to COVID-19** CHWs provided COVID-19-related information to the community [SR-7] **An essential package of services** Campaigns promoting care-seeking behaviour were also implemented [SR-13]	**Surveillance enabling the timely detection of shocks and their impact** Digitization of MCH records to further harmonize real-time data generation [SR-13] **Effective communication and flow of information** Community-level actions undertaken. This included awareness generation through leaders and peers to reduce fear and combat misinformation about the pandemic [SR-13]	**Adequate stock of essential equipment and supplies** Monitored the stock of PPE and essential drugs through the Central Medical Supply of the Ministry of Health [SR-13]
22	**Mexico**		NIL	NIL	NIL	**Adoptive/transformative capacity** Adoption of national guidelines for safe maternal healthcare [SR-23] **Absorptive capacity** Preservation of activities of CHWs/VHWs related to maternal health [SR-23]	NIL	NIL
23	**Morocco**		**Adequate and effective leadership** Since the announcement of the epidemic in China, the Moroccan government has deployed an institutional and risk-based communication strategy without having had any COVID-19 cases notified in the country [SR-15] **Coordination of activities across government and key stakeholders** Political commitment endorsed at the highest level, model of organization and coordination of the response integrated all key sectors and considered all levels of intervention (central, regional and local) [SR-15] Ethical aspects were integrated into the policy and practices in terms of preparedness and response to the pandemic [SR-15]	NIL	**Motivated and well-supported workforce** Remote platforms were established to provide psychological support and counselling services to health professionals [SR-15]	**Adoptive/transformative capacity** Remote platforms were established to provide psychological support and counselling services to health professionals and citizens who developed certain disorders in the form of distress, depression or acute panic disorders resulting from fear or confinement [SR-15] **Evidence of services related to COVID-19** Management protocols were developed in collaboration with the Scientific, Technical, and Advisory Committee of the Ministry of Health for the management of COVID-19 and were regularly updated on the basis of new knowledge about the disease [SR-15] **Ability to deliver services safely** Treatment services were integrated into all care structures and offered at home when the indication was justified [SR-15]	**Effective communication and flow of information** Multiple awareness-raising spots on preventive measures were produced and material distributed continuously to raise awareness to avoid the risk of contamination [SR-15]	**Adequate stock of essential equipment and supplies** Medicines and other pharmaceutical products were mobilized very early [SR-15]
24	**Mozambique**		**Adequate and effective leadership** The government issued national-level policy guidance related to RMNCH service continuity [SR-20]	NIL		**Transformative/adoptive capacity** Reorganization of space for service provision: reduced number of patients per day, reduced follow-up visits, reorganized maternity wards to maintain an appropriate distance between women in labour and to have a dedicated room for pregnant women with COVID-19 symptoms Vaccination was offered in more open spaces, and only small groups were allowed in the health facility at any one time [SR-2] **Evidence of service related to COVID-19** Establishment of new public health laboratories and linkages with private (including research) laboratories [SR-16]	**Effective communication and flow of information** A WhatsApp account enabled individuals to send a message saying Olá (‘hello’ in Portuguese) to a particular number and get updated information about local COVID-19 case rates [SR-16]	NIL
25	**Myanmar**		NIL	NIL	**Capacity building of health workers** An online training session was conducted for volunteers through video conferencing on volunteers’ psychosocial support for pregnant mothers, newborns, children, older people, and survivors of gender-based violence (GBV) [SR-12] **Appropriate level and distribution of human resources** Task shifting of staff [13]	**Adoptive/ transformative capacity** Pre-scheduled appointments were held for outpatient services, hospital based, and for immunization [SR-12] Free teleconsultations and information on FP and sexual health provided by non-governmental organization (NGO) partner [SR-12] Teleconsultations and hotlines carried out for older people’s health, alongside the provision of psychological support [SR-12] **Evidence of service related to COVID-19** ANC services, PMTCT testing, weighing, immunization, and iron supplements provided for pregnant women in quarantine centres [SR-12] **Ability to deliver services safely** IPC standards implemented in health facilities [SR-12] **An essential package of services** Ministry of Health and Sports website provided messages on menstrual hygiene, self-care, and nutrition, and hosted frequently asked questions for pregnant and lactating mothers and newborns/infants (Myanmar) [SR-12]	NIL	**Adequate stock of essential equipment and supplies** Additional supplies of self-injecting contraceptive (DMPA-SC) to prevent stock-outs introduced [SR-12]
26	**Nepal**		**Adequate and effective leadership** Interim guidance for SRMNCH services during the COVID-19 pandemic developed by the Ministry of Health and Population was approved and rolled out [SR-13]	NIL	**Motivated and well-supported workforce** Online portal for providing mental health and psychological support to healthcare providers [SR-12] **Appropriate level and distribution of human resources** Additional workforce for health acquired and health workforce reorganized to bring services to a normal level [SR-13]	**Adoptive/transformative capacity** Regional guidelines on the continuity of SRMNCAH services have also been used. These guidelines covered services for women, newborns, children and adolescents, including GBV [SR-13] Helplines were operationalized for reporting GBV and/or domestic violence [SR-12] Adolescents were provided with psychosocial counselling and online counselling regarding adolescent sexual and reproductive health [SR-12] **Ability to deliver services safely** IPC standards implemented in health facilities [SR-12] **Essential packages of services** RMNCAH information in the local catchment area was provided via public media and mobile text messages for sexual and reproductive health (SRH) awareness [SR-12]	**Surveillance enabling the timely detection of shocks and their impact** Continuous monitoring and assessment were used for evidence‐based planning and response [SR-13]	**Adequate stock of essential equipment and supplies** FP forecast was adjusted to anticipate a surge in migrant returnees [SR-12] Procurement of FP commodities [SR-12] Increased supply of FP commodities at the PHC level in partnership with private entities and NGOs [SR-12]
27	**Nigeria**		**Adequate and effective leadership** President signed the law for COVID-19 Health Protection Regulations 2021 [SR-14] **Coordination of activities across government and key stakeholders** The National Primary Health Care Development Agency (NPHCDA), multisectoral partners and stakeholders, and donors collaborated closely to develop a plan for continuing and optimizing PHC services during the COVID-19 pandemic [SR-9]	**Ensuring sufficient monetary** **resources in the system and** **flexibility to reallocate and inject extra funds into the system** Nigeria scaled up the delivery of social assistance to mitigate the financial impact of the pandemic on poor and vulnerable households This involved increasing coverage of the National Social Safety Nets Project (NASSP) routine cash transfer program [SR-9] **Purchasing flexibility and reallocation of funding to meet changing needs** Loans and importation of duty waivers granted to pharmaceutical firms for immunization [SR-9]	**Recruitment of health workers** Additional front-line health workers recruited and trained to provide RMNCAHN services [SR-12] **Motivated and well-supported workforce** The government provided incentive pay for health workers [SR-12]	**Adoptive/transformative capacity** The service delivery structure was reorganized to be responsive to the demands of the lockdown period. In some facilities, MNCH care, which was previously delivered to service users using a batched system on specific days, was adapted to occur on a rolling basis [SR-17] The government also procured trucks for TB case finding, targeting homes in TB hotspots and remote locations [SR-9] Proactive and mobile approaches to immunization, a mobile and outreach scheme for PHC, was introduced (e.g. I-MOP) [SR-9] Nigeria’s CDC integrated an interactive chat box into existing technology being used for communication [SR-16] Text messaging to provide psychosocial support and mental wellness for families [SR-12] **Essential packages of services** Innovations such as the mobile itinerant diagnostic facility (WoW truck), which provided diagnostic and treatment services for patients with TB, were introduced [SR-09] **Ability to deliver services safely** Delivered medications, non-pharmaceutical equipment, and supplies to people at home living with chronic conditions, thus protecting them from potential exposure at primary care facilities and in public transport [SR-24] **Evidence of service related to COVID-19** Screening, triage, and isolation for patients with COVID-19 [SR-12]	**Surveillance enabling the timely detection of shocks and their impact** Surveillance modernization: the Nigeria CDC deployed the Surveillance Outbreak Response Management and Analysis System, which supports case-based digital surveillance from health facilities [SR-16] **Effective communication and flow of information** Broadcast messages and awareness raising by NGOs)/community groups on accessing essential RMNCAH + N services and immunization [SR-12]	**Adequate stock of essential equipment and supplies** A dashboard for FP was developed to monitor and distribute adequate supplies of contraceptive commodities [SR-12] The Nigeria CDC established a centralized warehouse from which it distributed supplies to laboratories, treatment centres and the health ministry [SR-16]
28	**North Macedonia**		**Coordination of activities across government and key stakeholders** Coordinated effectively within (horizontally) and across (vertically) levels of government for developing new entities, such as special government emergency committees [SR-4]	**Ensuring sufficient monetary resources in the system and flexibility to reallocate** Complemented government funds with humanitarian aid [SR-04]	NIL	NIL	NIL	NIL
29	**Pakistan**		NIL	NIL	**Appropriate level and distribution of human resources** Redeployment of personnel from departments with low patient load to RMNCAAH departments [SR-12] Mobilized previously non-active female doctors for digital delivery of RMNCAH services through private partnerships [SR-12] **Capacity building of health workers** Training for health providers using distance learning/e-courses (Pakistan) [SR-12]	**Adoptive/transformative capacity** Routine and non-serious cases declined and referred to digital platforms [SR-12] Expanded Program on Immunization (EPI) services delivered by motorbikes and vans and door-to-door visits and counselling for child vaccination (routine immunization) [SR-19 & SR-12] Mobile phone apps, WhatsApp for accessing RMNCAH services, that is, telehealth consultations for maternal and newborn care in cities [SR-12] **Ability to deliver services safely** Delivery of contraceptives to homes, door-to-door medical abortion counselling, and FP commodities [SR-12]	**Effective communication and flow of information** Public broadcast channels, social media (Instagram), and health partners relayed public messages on COVID-19 protection and SRH, safe motherhood, self-care for pregnant women, nutrition, and GBV (Pakistan) [SR-12]	**Stock of PPE** Availability of PPE helped ensure the provision and utilization of maternal services, particularly delivery services [SR-19]
30	**Rwanda**		**Coordination of activities across government and key stakeholders** National COVID-19 Joint Task Force and COVID-19 prevention task force were setup [SR-14] **Adequate and effective leadership** Control guidelines were issued [SR-14]	NIL	NIL	**Adoptive/transformative capacity** Adopted new technologies to address difficult issues. For example, in Rwanda, drones were flown above houses in Kigali and remote areas to increase awareness of the COVID-19 pandemic and communicate prevention messages in the communities [SR-14] **Evidence of service related to COVID-19** People with COVID-19 and contacts in home isolation received text message check-ins via SMS for 2 weeks, enabling self-reporting of new symptoms or issues [SR-16]	NIL	NIL
31	**Russian Federation**		NIL	NIL	**Motivated and well-supported workforce** Financial compensation to support the health workforce in the form of one-time bonus payments [SR-04]	NIL	NIL	NIL
32	**Senegal**		**Coordination of activities across government and key stakeholders** Coordinated multi-sectoral response: Senegalese response, where public health and economic strategies were seen as inseparable (Senegal) [SR-14]	**Ensuring sufficient monetary resources in the system and flexibility to reallocate and inject extra funds into the system** Budget was made available for the resilience package and allocated to the health system to improve testing, treatment, tracing and prevention, and to enable the recruitment of additional health workers [SR-14]	**Recruitment of health workforce** Recruitment of 1500 additional health workers, especially in rural districts [SR-14]	NIL	NIL	NIL
33	**Serbia**		**Coordination of activities across government and key stakeholders** Coordinated effectively within (horizontally) and across (vertically) levels of government by establishing Operational Intersectoral Headquarters [SR-4]	**Ensuring sufficient monetary resources in the system and flexibility to reallocate** Complemented government funds with humanitarian aid [SR-04]	NIL	NIL	NIL	NIL
34	**Sierra Leone**		NIL	NIL	NIL	**Adoptive/transformative capacity** Adoption of national guidelines for safe maternal care [SR-23] **Absorptive capacity** Preservation of activities of CHWs/VHWs related to maternal health [SR-23] **An essential package of services** Implementation of communication campaigns [SR-23]	NIL	NIL
35	**South Africa**		NIL	NIL	**Appropriate level and distribution of human resources** Suspension of redeployment/rotation of staff in obstetric or newborn/neonatal facilities, wards, or clinics to other wards/clinics [SR-12] **Motivated and well-supported health workers** Psychosocial support and information for staff through Health-Connect app/Health Worker Alert [SR-12]	**Absorptive capacity** CHWs provided home visits for follow-up of patients with HIV, TB, mental disorders, postnatal care, and palliative care needs [SR-24] **Adoptive/transformative capacity** Virtual communications technology was used by the National Department of Health (NDOH) to support and provide technical guidance to provinces, partners, and front-line workers [SR-12] Mom-Connect (NDOH mobile health program for ANC and maternal health) platform linked to other services for early ANC booking, reminder messaging, facility status information, and referrals [SR-12] **Ability to deliver services safely** Delivered medications, non-pharmaceutical equipment and supplies to people at home living with chronic conditions, thus protecting them from potential exposure at primary care facilities and in public transport [SR-24] **Evidence of service related to COVID-19** Establishment of new public health laboratories and linkages with private (including research) laboratories [SR-16] **An essential package of services** Public health education campaigns that were carried out through community organizations and social media [SR-24]	**Surveillance enabling the timely detection of shocks and their impact** South Africa’s National Institute for Communicable Diseases worked with the National Department of Health to develop COVID-Connect and the COVID Alert South Africa app notification systems to provide results and notify people if they were in close contact with a patient diagnosed as positive [SR-16] **Effective communication and flow of information** Broadcast and social media campaign for health messaging on breastfeeding and service continuity for immunization, child health and nutrition, SRH, and ANC [SR-12]	**Adequate stock of essential equipment and supplies** Enhanced monitoring of vaccination supplies [SR-12]
36	**Sri Lanka**		**Adequate and effective leadership** Developed many guidelines, such as the Preparedness and Response Plan, rational use of PPE, hospital preparedness, and Stakeholder Engagement Plan (SEP) [SR-13]	NIL	**Appropriate level and distribution of human resources** Measures such as relocation of staff for specialized MNCH care in designated hospitals and safeguards to strengthen health workforce protection were put in placed [SR-13] **Capacity building of health workers** Staff were also trained on the use of PPE [SR-13]	**Adoptive/transformative capacity** Provided care in the ‘new normal’ phase (including high-risk approach in antenatal care, growth monitoring and immunization services, PPE and protective measures as well as implementation of national technical guidelines and reorganization of clinic services) [SR-13] Emergency referral transport for women, newborns, children, and adolescents was provided through a government-funded ambulance service [SR-13] **Ability to deliver services safely** Care of children under 5 years of age with illnesses such as diarrhoea, pneumonia, and severe acute malnutrition at the primary care level was continued with IPC measures and a reduction in the number of visits [SR-13]	**Effective communication and flow of information** The country used mass communication (including a social media campaign) and roped in the mass media to create awareness about MNCH [SR-13]	**PPE and other essential supplies and equipment for PHC service delivery** Equipment and supplies for EmONC care and supplies to protect personal hygiene were mobilized [SR-13]
37	**Sudan**		NIL	NIL	**Appropriate level and distribution of human resources** Redeployment of health workers [SR-12] **Recruitment of health workforce** Temporary recruitment of health workers [SR-12] **Motivated and well-supported workforce** Provision of incentives and staff retention in selected areas [SR-12]	**Adoptive/transformative capacity** Telemedicine, e-health, and m-health hotlines were established, i.e., hotline/call centre for FP and virtual consultations and the use of WhatsApp to send results and prescriptions [SR-12] **Evidence of service related to COVID-19** Screening, triage and isolation for patients with COVID-19 [SR-12]	NIL	**Adequate stock of essential equipment and supplies** Reinforcement and adaptation of supply chains, procurement, and logistics arrangements [SR-12]
38	**Tajikistan**		NIL	NIL	**Motivated and well-supported workforce** Provision of psychosocial support in the community for CMWs [SR 12] **Capacity building of the health workforce** Training on IPC protocols and measures for health workers and CHVs [SR 12]	**Adopted/transformative capacity** Night shifts introduced in PHC facilities [SR-12] ANC visits to health facilities were reduced to a maximum of four during pregnancy, with health workers conducting home visits [SR-12] **Evidence of service related to COVID-19** Ministry of Health and Social Protection ensured regular home visits for early detection of COVID-19, registration of pregnancies, and provision of ANC and other services [SR-12] **Ability to deliver services safely** IPC standards implemented in health facilities [SR-12]	NIL	**Personal protective equipment (PPE) and other essential supplies and equipment for PHC service delivery** PPE for health workers, and IPC in all health facilities [SR-12]
39	**Thailand**		**Adequate and effective leadership** Developed guidelines to protect essential SRMNCAH services during emergencies [SR-13]	NIL	**Capacity building of health workers** Offered continuous training for health service providers on SRMNCAH [SR-13]	**Adoptive/transformative capacity** Expanded the role of VHVs for surveillance and generated community capacity for resilience response [SR-7] MCH including FP and birth control services at the community level through VHVs conducting visits to families, providing information and referrals, and monitoring the risk of COVID-19. They also facilitated and sent public health information from the community to public health officers [SR-13]	NIL	NIL
40	**Timor-Leste**		**Adequate and effective leadership** MoH developed specific clinical protocols and guidelines for ANC and intrapartum and immediate postpartum care [SR-13] **Coordination of activities across government and key stakeholders** Country preparations and responses to the pandemic (included strengthening of the health system, isolation rooms, laboratory facilities, and PPE distribution as well as encouraging preventive measures, and reactivation of RMNCH working group) [SR-13]	NIL	NIL	**Absorptive capacity** Maternal and newborn health catch-up service provision through comprehensive outreach activities and pre-scheduled appointments [SR-12] **Adoptive/transformative capacity** All SRMNCAH services were provided at all levels. Family planning services and methods, routine immunization for children under 5 years of age, and nutrition counselling and growth monitoring of children under 5 years of age were continued at the community level through community workers and mobile clinics [SR-13] Modified Integrated Medical Outreach Programme that enabled immunization and contraceptive access and general health care services for mothers and young children in rural and remote areas [SR-12] **Evidence of service related to COVID-19** Establishment of a separate maternity isolation ward at the maternity clinic [SR-13]	NIL	**Personal protective equipment (PPE) and other essential supplies and equipment for PHC service delivery** PPE for health workers, and IPC in all health facilities [SR-12]
41	**Uganda**		**Adequate and effective leadership** The government issued national-level policy guidance related to RMNCH service continuity [SR-20]	NIL	**Motivated and well-supported workforce** Transport for health workers organized where needed; improvisation of overnight lodging facilities for health workers in some districts [SR-12] Daily risk allowance for health workers was offered [SR-12] **Capacity building of the health workforce** Training of trainers in essential health services [SR-12]	**Absorptive/transformative capacity** FP services were integrated into other health services, including postpartum, young child, and antiretroviral therapy (ART) clinics, PNC also integrated into EPI outreach [SR-12] CHWs provided home visits for follow-up of patients with HIV, TB, mental disorders, postnatal care and palliative care needs [SR-24] Special child clinics for MCH and HIV services to reduce congestion [SR-12] Psychosocial and ART adherence counselling via phone and WhatsApp for adolescents and mothers, and text message appointment reminders [SR-12] **An essential package of services** Community outreach activities for adolescents regarding SRH, HIV counselling and testing, ANC, contraception, condom distribution, and human papillomavirus immunization [SR-12] **Ability to deliver services safely** Delivered medications, non-pharmaceutical equipment, and supplies to people at home living with chronic conditions, thus protecting them from potential exposure at primary care facilities and on public transport [SR-24] **Evidence of service related to COVID-19** People with COVID-19 and contacts in home isolation received text message check-ins via SMS for 2 weeks, enabling self-reporting of new symptoms or issues [SR-16]	**Surveillance enabling the timely detection of shocks and their impact** Surveillance modernization: Uganda shifted to the use of the Go-Data tool; managed by the WHO Global Outbreak and Response Network and included secure data exchange and functionality for case investigation, contact follow-up, and visualization of chains of transmission [SR-16] **Effective communication systems and flow of information** Many telecommunications companies incorporated public health messages into their routine services. In Uganda, MTN and Airtel disseminated messages about how to keep safe from COVID-19; [SR-16]	**Personal protective equipment (PPE) and other essential supplies and equipment for PHC service delivery** PPE for health workers, and IPC in all health facilities [SR-12]
42	**Ukraine**		**Coordination of activities across government and key stakeholders** Coordinated effectively within (horizontally) and across (vertically) levels of government for instance created new entities, such as special government emergency committees [SR-4]	**Ensuring sufficient monetary resources in the system and flexibility to** Secured additional funding through loans and grants. (by financial assistance and through additional funding through debt service relief from international lending institutions such as the International Monetary Fund (IMF)) [SR-4]	**Motivated and well-supported workforce** Financial compensation to support the health workforce in the form of one-time bonus payments [SR-04]	NIL	NIL	NIL
43	**Yemen**		NIL	NIL	**Capacity building of health workers** Training on IPC protocols and measures for health workers and community health volunteers. [SR 12] Provision of psychosocial support in the community for CMWs [SR-12] **Motivated and well-supported workforce** Health workers are retained with incentives to prevent health system collapse due to a reduction in donor support [SR-12]	**Adoptive/transformative capacity** Hotline services for FP, ANC, and support for women and girls at risk of/experiencing violence (e.g. WhatsApp for medical consultations including obstetrics/gynaecology) [SR-12] Hotline service for older people on general health, COVID-19 prevention, and chronic diseases [SR-12] Mid-upper arm circumference (MUAC) measurement was established as the sole criterion in assessing nutritional status and the basis of entry criteria to outpatient therapeutic/supplementary feeding programs [SR-12] Modification of entry and follow-up criteria to outpatient therapeutic/supplementary feeding programs for pregnant and lactating mothers with moderate acute malnutrition [SR-12] **An essential package of services** Awareness-raising activities on FP, maternal, newborn, and child services, GBV and nutrition services specifically targeting migrants and displaced persons (Yemen) [SR-12] **Ability to deliver services safely** Mobile units conducted examinations for physical and mental health among children in wake of school closures [SR-12]	**Effective communication and flow of information** Communication related to FP service availability through national broadcasting channels and community volunteer efforts [SR-12]	**Adequate stock of essential equipment and supplies** Construction and rental of additional warehouses for storing additional supplies [SR-12] Availability of additional supplies of medical commodities (FP, reproductive health kits, clean birth kits, medicines for treating rape and post-abortion care [SR-12]
44	**Zimbabwe**		**Adequate and effective leadership** The government issued national-level policy guidance related to RMNCH service continuity [SR-20]	NIL	NIL	**Adoptive/transformative capacity** A mobile outreach model was launched that integrated community-based outreach with facility-based outreach [SR-21} **Absorptive capacity** CHWs provided home visits for follow-up of patients with HIV, TB, mental disorders, postnatal care, and palliative care needs [SR-24] Mop-up campaigns for immunization services to reach out to children who missed their appointments due to COVID-19 [SR-20] **Ability to deliver services safely** Delivered medications, non-pharmaceutical equipment and supplies to people at home living with chronic conditions, thus protecting them from potential exposure at primary care facilities and in public transport [SR-24] **An essential package of services** Public health education campaigns that were carried out through community organizations and social media [SR-24]	NIL	NIL

The scoping review was reported according to the Preferred Reporting Items for Systematic Reviews and Meta-Analyses extension for Scoping Reviews (PRISMA-ScR) Checklist [23] (Table 8).

### Patient and public involvement

There has been no patient or public involvement in the development of this protocol, neither in the scoping review itself.

### Coordination, monitoring & quality control

The study has ensured quality control by adhering to the standard protocols for conducting the scoping review. This includes the use of the PRISMA-ScR checklist for reporting review findings. Furthermore, this review was undertaken by two well-experienced researchers with expertise in the scoping review. The reviewers were oriented on the conduct of the scoping review to ensure consistency in the data collection and analysis approach. In addition, throughout the review process (study selection, data extraction process, and data analysis) frequent debriefing meetings were carried out within the research team to ensure consensus among the team.

## Results

### Characteristics of studies

Our findings encompassed a total of 26 studies and documents across 44 LMICs, which were published between the years 2020 and 2021. The included articles document any aspect related to the six health systems building blocks of PHC services (health services delivery, health workforce, governance, finance, supplies and equipment and information system) or the combination of these during the COVID-19 pandemic. The distribution of different types of publications in our analysis includes reports/updates (*n* = 13), journal articles (*n* = 8), policy briefs (*n* = 2), reviews (*n* = 2), and guidance documents (*n* = 1). The overall results were synthesized according to the WHO health systems building blocks framework (Additional file 2). The country-specific key findings are presented in Table 5.

#### Governance

In the context of our study, governance refers to the comprehensive management, leadership, and decision-making processes that guided the policies, strategies and operations of the PHC system during the COVID-19 pandemic.

#### Adequate and effective leadership

Adequate and effective leadership plays a critical role in building a resilient health system during a crisis. Leaders in healthcare organizations and government agencies are responsible for strategic decisions to enable healthcare systems to withstand the challenges posed by the pandemic. Out of the 44 LMICs, only 16 countries (Bangladesh, Bhutan, Bosnia and Herzegovina, Ethiopia, India, Kenya, Morocco, Mozambique, Nepal, Nigeria, Rwanda, Sri Lanka, Thailand, Timor-Leste, Uganda and Zimbabwe) introduced guidelines and response strategies that reflected adequate and effective leadership during COVID-19 pandemic (Table 6).

**Table 6:** Country summary table

S.NO	Countries	Governance		Financing		Health workforce			Service delivery				Information system		Supplies and equipment
		Adequate and effective leadership	Coordination of activities across government and key stakeholders	Ensuring sufficient monetary resources in the system and flexibility to reallocate funds	Purchasing flexibility and reallocation of funding to meet changing needs	Appropriate level and distribution of human resources	Motivated and well-supported workforce	Recruitment and capacity building of the health workforce	Absorptive, adoptive, and transformative capacity	An essential package of services	Evidence of service related to COVID-19	Ability to deliver services safely	Surveillance enabling the timely detection of shocks and	Effective communication and flow of information	PPE and other essential supplies and equipment for PHC service delivery
1	Albania						X				X				
2	Bangladesh	X	X			X	X	X	X	X	X		X	X	X
3	Bhutan	X		X		X		X	X			X		X	
4	Bolivia							X	X		X	X	X		X
5	Bosnia and Herzegovina	X					X								
6	Botswana								X	X		X			
7	Brazil					X		X	X	X					X
8	Bulgaria						X								
9	Cameroon								X		X	X		X	X
10	Congo					X		X	X					X	X
11	Ethiopia	X	X					X	X		X	X	X	X	X
12	Ghana								X	X	X	X	X	X	
13	Haiti								X						
14	India	X		X		X		X	X	X	X	X	X		X
15	Indonesia								X		X	X			
16	Kenya	X	X			X	X	X	X	X	X	X		X	X
17	Kyrgyzstan			X			X								
18	Lesotho								X						
19	Liberia								X	X					X
20	Malawi					X		X	X	X		X	X	X	
21	Maldives							X	X	X	X		X	X	X
22	Mexico								X						
23	Morocco	X	X				X		X		X	X		X	X
24	Mozambique	X							X		X			X	
25	Myanmar					X		X	X	X	X	X			X
26	Nepal	X				X	X	X	X	X		X	X		X
27	Nigeria	X	X	X	X		X	X	X	X	X	X	X	X	X
28	North Macedonia		X	X											
29	Pakistan					X		X	X			X		X	X
30	Rwanda	X	X						X		X				
31	Russian Federation						X								
32	Senegal		X	X				X							
33	Serbia		X	X											
34	Sierra Leone								X	X					
35	South Africa					X	X		X	X	X	X	X	X	X
36	Sri Lanka	X				X		X	X			X		X	X
37	Sudan					X	X	X	X		X				X
38	Tajikistan						X	X	X		X	X			X
39	Thailand	X						X	X						
40	Timor- Leste	X	X						X		X				X
41	Uganda	X					X	X	X	X	X	X	X	X	X
42	Ukraine		X	X			X								
43	Yemen						X	X	X	X		X		X	X
44	Zimbabwe	X							X	X		X			
Total		16	11	8	1	13	16	21	35	17	20	21	11	17	22

The Nigerian President approved and enacted the COVID-19 Health Protection Regulations in 2021. This law established a legal framework to protect public health and mitigate the spread of the COVID-19 pandemic within the country [24]. The national guidelines were introduced on maternal, newborn, and child health (MNCH) and family planning (FP) in Bangladesh, while countries such as Bhutan, India, Kenya, Mozambique, and a few other states also developed guidelines on MNCH with an element of reproductive component nested into it [24–30] (Table 5). In Timor-Leste, protocols for delivering antenatal care (ANC) and postpartum care in the context of the COVID-19 pandemic were developed and implemented [28]. Furthermore, since the announcement of the epidemic in China, the Moroccan government deployed an institutional and risk-based communication strategy without any COVID-19 cases notified in the country [31].

#### Coordination of activities across government departments and among key stakeholders

Coordination of activities across various departments in the government and key stakeholders ensures a comprehensive and coordinated response to the pandemic, including prevention, detection of cases and response efforts. However, evidence of coordination and collaboration across the different institutes and governmental organizations to tackle the crisis while maintaining essential healthcare services emerged only from 11 countries (Bangladesh, Ethiopia, Kenya, Morocco, Nigeria, North Macedonia, Rwanda, Senegal, Serbia, Timor-Lester and Ukraine) [1, 8, 24, 28, 31–33] (Table 6). For instance, the Ethiopian Public Health Institute (EPHI) initiated a program called COVID -19 Plan Revitalization Movement, which aimed to bolster the response to the COVID-19 pandemic by raising public awareness through risk communication and community involvement [SR-14]. Another such example was from Morocco, where political commitment, organization and coordination of the response ensured the integration of all key stakeholders across all levels (central, regional and local) [31]. Similarly, in Nigeria, the key stakeholders inclusive of the National Primary Health Care Development Agency (NPHCDA) and other multisectoral partners collaborated to develop a plan for continuing and optimizing PHC services during the COVID-19 pandemic [33].

### Financing

Adequate financial resources during a crisis allow for better management of resources. Our analysis focussed on two financing strategies to ensure resource mobilization during a public health crisis. These are explained next.

#### Ensure sufficient monetary resources in the system and flexibility to reallocate and inject extra funds into the system

In total, 8 out of 44 countries (Bhutan, India, Kyrgyzstan, Nigeria, North Macedonia, Senegal, Serbia and Ukraine) had adopted strategies to ensure the availability of necessary monetary resources to respond to the pandemic effectively and efficiently without compromising essential healthcare services (Table 6). For instance, to reduce the financial toll of the pandemic on poor households, Nigeria expanded the reach of the National Social Safety Nets Project (NASSP) and its regular cash transfer initiative [33]. Another strategy was the mobilization of private sector funding to supplement existing stock in Bhutan [34].

Flexibility to reallocate and inject extra funds into the system refers to the ability to adjust the allocation of resources in response to the COVID-19 crisis [19]. This involved reallocating resources from one area of the system to another or injecting additional funding to address emerging needs during the COVID-19 pandemic. In Senegal, the budget was allocated to the health system as part of a resilience package aimed at improving testing, treatment, tracing, and prevention measures, alongside enabling the recruitment of additional health workers [24].

#### Purchasing flexibility and reallocation of funding to meet changing needs

Nigeria was the only country that granted loans and waivers to pharmaceutical firms to encourage the development of vaccines against COVID-19 [33] (Table 5).

### Health workforce

Having a strong, well-trained, and adequately deployed health workforce serves as the foundation to respond to the crisis.

#### Appropriate level and distribution of human resources

Ensuring adequate level and distribution of the health workforce remained a popular area of intervention among the LMICs during the COVID-19 pandemic. The evidence from 13 countries (Bangladesh, Bhutan, Brazil, Congo, India, Kenya, Malawi, Myanmar, Nepal, Pakistan, South Africa, Sri Lanka and Sudan) demonstrated a wide range of measures implemented to ensure sufficient stock of front-line healthcare workers to manage the influx of patients with COVID-19 (Table 6).

In Brazil, specialists were relocated to PHC centres to facilitate access for pregnant women [34]. Similarly, Congo redeployed health personnel from departments with low patient load to Reproductive, Maternal, Neonatal, Child, and Adolescent Health (RMNCAH) departments [34]. India also redeployed additional nursing staff to PHC centres from other facilities [34], while Kenya redeployed staff within and between facilities [25]. In Pakistan, inactive women doctors were mobilized to deliver RMNCAH services through virtual platforms in collaboration with the private health sector [34].

#### Motivated and well-supported workforce

Healthcare workers were under extreme mental and physical duress during the COVID-19 pandemic, and thus it was essential to provide them with adequate psychological support and to prevent burnout. A total of 16 countries (Albania, Bangladesh, Bosnia and Herzegovina, Bulgaria, Kenya, Kyrgyzstan, Morocco, Nepal, Nigeria, Russian Federation, South Africa, Sudan, Tajikistan, Uganda, Ukraine and Yemen) [1, 25, 28, 31, 34, 35] documented coping strategies for healthcare personnel (Table 6). The most frequently documented strategy was the provision of psychosocial support and counselling services to facilitate the health workforce to cope with the pandemic [34].

#### Recruitment and capacity building of the health workforce

A total of 21 LMICs (Bangladesh, Bhutan, Bolivia, Brazil, Congo, Ethiopia, India, Kenya, Malawi, Maldives, Myanmar, Nepal, Nigeria, Pakistan, Senegal, Sri Lanka, Sudan, Tajikistan, Thailand, Uganda and Yemen) had documented evidence on recruitment and capacity building of their healthcare workers during the pandemic (Table 6). It was crucial to enhance the capacity of the health workforce through training and preparing healthcare workers to cope with the increased work burden during the pandemic [1, 8, 24, 25, 28, 30, 31, 34–39]. One interesting finding that emerged during the synthesis of evidence was the use of digital technology to train healthcare workers. For instance, in Ethiopia, a mobile learning platform was introduced that utilized an interactive text message and voice response application for training the healthcare personnel on sexual reproductive, maternal, neonatal, child, and adolescent health (SRMNCAH) services during the COVID-19 pandemic [34].

Some LMICs also invested in training medical staff and postgraduate students to provide emergency obstetric care, family planning and antenatal care services [25, 26, 28, 30, 33, 34, 38, 40]. India expedited recruitment and filled existing staff vacancies amid the COVID-19 pandemic[38]. The involvement of community health workers (CHWs) and community health volunteers (CHVs) and their training across various PHC activities such as infection prevention and control in Yemen [34], training of Rohingya refugees (who worked as CHVs) in immunizations, management of non-communicable diseases, and maternal and child health support [37], and activities including preventing the spread of rumours and misinformation in the community by India [37] was evident in the review.

### Service delivery

Provision of safe and effective and quality healthcare services to individuals and communities while reorganizing the PHC infrastructure remains the hallmark of a resilient health system.

#### Absorptive, adoptive and transformative capacity

In 35 countries, evidence related to the three essential resilience capacities (1. absorptive, 2. adoptive, 3. transformative) of PHC systems was discussed (Table 6). The absorptive capacity refers to ‘the capacity of the healthcare system to respond to the pandemic while continuing to deliver the same level of basic healthcare services’ [41]. The example includes the development of contingency plans in Bhutan to ensure the provision of essential healthcare services and the activation of micro-plans for health facilities [28]. In Kenya, specific policies were implemented to guarantee uninterrupted essential care for expectant and nursing women [25]. The adoptive capacity of health is defined as ‘the adaptation to the shock (COVID-19 pandemic) and continue providing the same level of healthcare services with fewer or different resources’ [41]. The transformative capacity refers to ‘the ability of the health system to reorient and transform its functions in response to the shocks’ [41]. Examples of the later two categories include the strengthening of referral and transport mechanisms in Bolivia, India and Sri Lanka [28, 34]. Furthermore, in India, health facilities at all levels (block, district and state) were mapped, and healthcare service delivery was restructured to cater to both COVID-19 and non-COVID-19 care needs [42].

Transformation of service delivery was also evident with the use of digital platforms across multiple countries. The use of WhatsApp, Viber, hotlines, and telemedicine for providing maternal, child, reproductive, and adolescent health services were used in Bolivia and Bangladesh [34]. Refer to Table 5 (service delivery domain) with specific examples illustrating three different capacities while coping with the COVID-19 pandemic.

#### An essential package of healthcare services

Our evidence synthesis revealed that 17 out of 44 countries (Bangladesh, Botswana, Brazil, Ghana, India, Kenya, Liberia, Malawi, Maldives, Myanmar, Nepal, Nigeria, Sierra Leone, South Africa, Uganda, Yemen and Zimbabwe) implemented measures to ensure the delivery of essential public health services during COVID-19 (Table 6). This encompassed strategies that aimed to develop a package of services that was adequately resourced, organized and distributed by identifying vulnerable population groups (ensuring that appropriate data are collected) and ensured adequate access to services and evidence of continuing essential public health functions (including health education and awareness).

Uganda conducted community outreach activities for adolescents on sexual and reproductive health (SRH), HIV counselling and testing, ANC, contraception, condom distribution, and human papillomavirus immunization [34]. Brazil implemented an elderly health home record book to record personal, social, and family data, health conditions, health behaviours and vulnerabilities, and guidance on self-care [34].

#### Evidence of services related to COVID-19 pandemic

A total of 20 countries (Albania, Bangladesh, Bolivia, Cameroon, Ethiopia, Ghana, India, Indonesia, Kenya, Maldives, Morocco, Mozambique, Myanmar, Nigeria, Rwanda, South Africa, Sudan, Tajikistan, Timor-Leste and Uganda) reported evidence of measures taken by LMICs to provide services for COVID-19 testing, isolation and quarantine (Table 6). In Albania, PHC providers assisted public health surveillance teams with contact tracing [1]. In India, several measures were instituted, such as the introduction of standard operating procedures for screening, triage, and isolation for suspected maternal and newborn COVID-19 cases [34]. Furthermore, antenatal care services for pregnant women were offered at quarantine centres in Myanmar [34] (Table 5).

#### Ability to deliver services safely

Alongside ensuring the continuity of essential healthcare services to the population during COVID-19, patient and health worker safety is also crucial.

The evidence of safe delivery of services emerged from 21 countries during our review. This includes Bhutan, Bolivia, Botswana, Cameroon, Ethiopia, Ghana, India, Indonesia, Kenya, Malawi, Morocco, Myanmar, Nepal, Nigeria, Pakistan, South Africa, Sri Lanka, Tajikistan, Uganda, Yemen and Zimbabwe [24–26, 28, 30, 34, 35] (Table 6). For instance, in Bolivia, prenatal and PNC visits were scheduled to reduce the number of women in close contact at health facilities [34]. In Sri Lanka, while treating children under 5 years of age at the primary care level, infection prevention and control measures were followed [28]. (Table 5).

### Information system

The adaptations in the information system for timely data collection and evidence-based decisions drive the public health response during a crisis (Table 5).

#### Surveillance and timely generation of data during the COVID-19 pandemic

Surveillance played a critical role in facilitating the identification of COVID-19 cases, tracking the spread of the virus and informing public health interventions to control the outbreak [26, 30, 32, 42–44]. Surveillance measures were evident in 11 out of 44 countries, including Bangladesh, Bolivia, Ethiopia, Ghana, India, Malawi, Maldives, Nepal, Nigeria, South Africa and Uganda (Table 6). India managed the pandemic utilizing an integrated disease surveillance system [42]. Bolivia coordinated with RedBol12 and the National Sexually Transmitted Infection/HIV/AIDS program to support surveillance, information and referral centres [34]. In Nigeria, case-based digital surveillance from health facilities was carried out through the Surveillance Outbreak Response Management and Analysis System [43]. Similarly, South Africa developed COVID Connect and COVID Alert South Africa app notification systems for COVID-19 management [43] (Table 5). Furthermore, in the Maldives, monitoring of service data and digitization of maternal and child health records assisted in harmonizing real-time monitoring [28].

#### Effective information (communication) systems and flows

Evidence from 17 out of 44 countries (Bangladesh, Bhutan, Cameroon, Congo, Ethiopia, Ghana, Kenya, Malawi, Maldives, Morocco, Mozambique, Nigeria, Pakistan, South Africa, Sri Lanka, Uganda and Yemen) demonstrated measures to facilitate the effective flow of information across different institutions (Table 6). For instance, in Morocco, multiple awareness-raising spots on preventive measures have been produced and distributed continuously to raise awareness to avoid the risk of contamination [31]. Similarly, in Cameroon, broadcast, print and social media campaigns were utilized to raise awareness of the reopening of health facilities for routine healthcare services [34].

### Supplies and equipment

#### PPE and other essential supplies and equipment for PHC service delivery

The provision of PPE and other essential supplies and equipment for PHC service delivery was documented by 22 out of 44 LMICs (Table 6).

Evidence from Kenya, Maldives, Pakistan, and Sri Lanka revealed the availability of PPE being ensured in PHC facilities [24, 28, 45–48] to safeguard the continuity of health services [36]. The LMICS including Cameroon, Ethiopia, Maldives, Myanmar, Morocco, Nepal, Nigeria, South Africa, and Yemen documented measures to ensure the availability of essential medicines, equipment, and supplies at the facility level during the COVID-19 pandemic [24, 28, 31, 34, 43]. This includes strengthening the supply chain and procurement of RMNCAH commodities in Ethiopia [34], as well as the mobilization of equipment and supplies for emergency obstetric and newborn care (EmONC) care and personal hygiene in Sri Lanka [28]. In addition, a monitoring dashboard was developed in Nigeria to ensure appropriate distribution and availability of contraceptive supplies [34], while in Nepal the supply of FP commodities was increased at the PHC level through collaborations with private organizations and non-governmental organizations (NGOs) [34].

## Discussion

The unprecedented challenge posed by the COVID-19 pandemic was responded to by a variety of measures adopted by 44 LMICs around the world to continue uninterrupted healthcare services at the PHC level. To our knowledge, this scoping review is the first of its kind in consolidating the existing evidence across resource-constrained settings on the capacity of the PHC system during the COVID-19 pandemic by using the WHO’s six-building blocks framework. Despite being faced with significant challenges, such as limited funding, workforce shortages, weak and poor governance, and weak health systems, our review demonstrated that several LMICS implemented innovative strategies and transformed the PHC infrastructure to maintain the delivery of PHC services, showcasing their ability to be resilient in times of crises.

Our review identified commonalities among countries in their response to fight the pandemic and initiation of public health measures that fall under the larger ambit of PHC. In general, the majority of LMICs implemented measures and strategies across the domains of governance, service delivery, and health workforce. The role of governance and leadership to coordinate the PHC response system during the pandemic has emerged during our review. The governing bodies of LMICs unequivocally prioritized healthcare services for SRMNCH during the COVID-19 pandemic over other services [28]. The response was led by the central and local health governing bodies that introduced national-level guidelines and protocols for SRMNCAH in multiple countries including Bangladesh, Bhutan, India, Kenya, and Mozambique, among others [8, 24, 26, 28, 29]. The measures introduced at the central and local governance trickled down to the service delivery level, which exhibited the shock absorptive capacities of LMICs to respond to the pandemic while continuing to deliver essential healthcare. For example, in Kenya, special guidelines were enacted to ensure that pregnant and breastfeeding women continued to receive the requisite care without any disruption [30]. Likewise, India continued offering both non-COVID-19 and COVID-19-related health services by decentralizing essential healthcare services [42]. (Table 5). Countries including Bolivia, India, and Sri Lanka strengthened referral systems and transportation facilities [28, 34], with evidence of emergency referral transport systems established for women, children and adolescents as reported from these countries. (Table 5). Contingency planning remains a crucial process in being prepared with needed resources in the pre-disaster phase. We found an evidence of contingency planning only in Bhutan, which involved ensuring the provision of essential healthcare services and the development of micro-plans for service provision at health facilities [28] (Table 5).

LMICs also adopted innovative and technologically driven strategies to reorient their PHC services during the COVID-19 pandemic; their approach towards using and integrating digital options, however, varied across multiple countries. Nigeria utilized a surveillance outbreak response management and analysis system to support case-based digital surveillance [43], while Bolivia took the opportunity of the digital platform to build health workers’ capacity [34]. The use of the WONDER App (mobile application) in India was unique to tracking high-risk pregnancies and continuity of care [34](Table 5). Furthermore, the use of WhatsApp and other telemedicine for providing maternal, child, reproductive and adolescent health services was documented in Bolivia and Bangladesh [34]. Additionally, examples of the use of digital technologies kept flourishing in LMICs amidst the COVID-19 pandemic. Some high-income countries such as China and South Korea reported the use of artificial intelligence to develop faster and more accurate COVID-19 diagnostic tests and to analyse large amounts of data to identify patterns and predict the spread of the virus [49]. Though transforming the PHC service delivery with digital options are appreciated, what is important is to also investigate how well the quality of healthcare services was ensured and whether countries were able to sustain the digital options in PHC service delivery. These, however, remain beyond the scope of our work.

Our review also exhibited that several countries grappled with a shortage of healthcare workers, which made it even more challenging for healthcare systems to cope with the influx of patients, resulting in long wait times, delayed care, and in some cases, inadequate care [1, 24, 30]. To address this challenge, many countries recruited and trained additional health workers and provided incentive pay to retain and motivate them [1, 24, 30, 31, 35] (Table 5). The mobilization of CHWs and their capacity building was also evident in a few of the countries (Nigeria, Malawi, Bangladesh, Yemen, India) to offer services for COVID-19 and other essential healthcare services. A similar approach was documented in developed countries such as Germany, where HCWs were incentivized using bonus payments and rewards during the COVID-19 pandemic. Though monetary incentives have a strong influence on health workers' motivation and retention [49], resource-constrained countries must plan sustainable financing schemes for health worker retention in the longer run.

To build resilient health systems, it is crucial not only to maintain essential services, but also to ensure that COVID-19 cases can be managed at the PHC level. Our scoping review revealed that despite facing numerous challenges, most LMICs developed sophisticated systems for screening, triaging and diagnosing COVID-19 at the PHC level while maintaining the provision of essential services [1, 34]. Our findings are consistent with those of Haldane et al., who documented measures adopted by countries including active surveillance, testing, contact tracing, use of innovative and digital solutions, and preservation of PHC services by supporting CHWs for outreach services [49].

Our review of the 44 LMICs revealed that Nigeria and India were the only countries that exhibited evidence of resilience measures across all six health systems building blocks (Table 6). With the emergence of the COVID-19 pandemic, the Nigerian President signed the law for COVID-19 Health Protection Regulations 2021 to safeguard the population’s wellbeing by enforcing mandatory compliance with facility protocols [24]. Under this law, a designated space or holding bay was established at all facility levels (primary, secondary and tertiary) for the initial triage of individuals suspected to have COVID-19 [24, 34]. The National PHC Development Agency collaborated with multisectoral partners and donors to develop a plan for continuing and optimizing PHC services that could be scaled up and sustained over time [33]. Additionally, the Nigerian government recruited, trained and incentivized healthcare workers to continue the provision of essential services [34].

Our analysis revealed that only a few of the low-resource countries struggled to address the healthcare financing measures for the continued delivery of PHC services amidst the COVID-19 pandemic [24, 28]. Noteworthy to mention is the budget allocation to the health system in Senegal (part of the resilience package for diagnostic and treatment services for COVID-19), the launch of an insurance package for the health workforce in India, and the purchasing flexibility and duty waivers to pharmaceutical companies in Nigeria for vaccine development [24, 28] (Table 5).

## Strengths and limitations

To the best of our knowledge, this is the first scoping review that has described the resilience of the LMICs’ PHC systems during the COVID-19 pandemic. The use of the WHO health systems building blocks framework integrated with other resilience frameworks facilitated in collating and compiling the overall resilience-enhancing activities (Additional file 3).

The challenges encountered during the conduct of the scoping review included: (i) reporting of measures and interventions at the health systems level rather than at the PHC level, which led to discussions in our team to sort and segregate measures specific to the PHC level; (ii) overlapping elements across the building blocks framework leading to duplication of results in a few areas, which led us to revisit the framework and take another look at the analysis; and (iii) the results synthesized and recommendations emerging from our review are based on the findings obtained. Therefore, any resilience measures by LMICs in our review could have been potentially missed in the case that it was not documented (Additional file 4).

The 44 countries that were included in our analysis represent diversified healthcare systems, with a mix of public and private healthcare actors and limited resources with a wide range of contextual factors that may have potentially affected countries’ approaches to fight against the COVID-19 pandemic. Understanding the contextual challenges (pre-pandemic as well as during the pandemic) experienced by these territories is instrumental in understanding the resilience measures that we were trying to synthesize. However, during data extraction, it was realized that not all countries reported challenges and limitations specific to the strategies and measures to continue to deliver PHC services. Hence, this limited our approach to documenting country-specific challenges. Some of the common challenges that were reported for 17 out of 44 countries included in our review (Bolivia, Brazil, Kazakhstan, Tajikistan, Pakistan, Sudan, Yemen, Democratic Republic of Congo, Ethiopia, Nigeria, South Africa, Uganda, Bangladesh, India, Myanmar, Nepal and Timor-Leste) include disrupted information systems, balancing the secondary and tertiary care services in hospitals with PHC services, limited transport to women and children, and re-purposing health workers to COVID-19, to mention a few [34].

A critical insight into country-specific contextual factors was thus beyond the scope of this review, as we primarily assessed the resilience strategies adopted by countries for the PHC system amidst the COVID-19 pandemic. How countries’ approaches were challenged and restricted by contextual factors require a next level of analysis. 

## Conclusions and recommendations

The COVID-19 pandemic presented an opportunity for countries to act as catalysts for action to offer uninterrupted healthcare services. Commonalities and differences were found in countries’ approaches and use of strategies for offering an interrupted healthcare service delivery during the pandemic. Resilience across LMICs was demonstrated by a variety of strategies, including releasing national guidelines for healthcare service delivery, task-shifting measures to community-based health workers, utilizing digital platforms for healthcare service delivery, building capacity of health workers, and investing in public health surveillance to the mobilization of private health sector funding, to mention a few.

To strengthen the resilience of the PHC system during crises, recommendations include: (i) prioritizing community-based PHC planning, budgeting, staffing, optimizing surge capacity of the health workforce, and other dimensions of health systems strengthening, as it can play a critical role in any emergency response; (ii) establishing and strengthening the communicable disease monitoring and surveillance system at various levels (community surveillance, integrated disease surveillance, etc.), as it is essential to facilitate timely response for the continuity of essential health services and to monitor the population’s health during the crisis; and (iii) strengthening the cross-sectoral response at the country level to offer a more coordinated public health response during a pandemic of such scale.

As only a few countries demonstrated the availability of necessary monetary resources to respond to the pandemic, there is therefore a need to (iv) build on the capacities of public health managers, policymakers, and PHC managers in financial risk planning to ensure the availability of needed financial resources during a public health emergency. Realizing that the use of digital technologies accelerated during the COVID-19 pandemic to offer essential healthcare services. Therefore, moving forward, LMICs must (v) invest to strengthen their technical competencies in providing healthcare services using digital platforms such as blended training programs, use of telemedicine, teleconsultation, etc., to continue the execution of PHC services during a public health crisis.

All five recommendations addressed above are practical measures that could potentially result in mid-term to long-term sustainability in countries provided they are part of the countries’ strategic planning and executed with a diverse and strong team alongside responsive health governance.

### Supplementary Information

**Additional file 1. **Data extraction sheet

**Additional file 2. **Analysis

**Additional file 3. **ERC Letter

**Additional file 4. **Corresponding references

## Data Availability

The datasets used and/or analysed during the current study are available from the corresponding author on request.

## Annexure

See Tables 7 and 8

**Table 7:** Data extraction sheet

Literature code	Publication date	Source/citation	Title	Country	Publication type	WHO building block(s)	Targeted services	**Health setting**	**Main outcome/impact**	**Strategies adopted by countries & key recommendations/lessons learned**
SR-1	Dec 2021	https://data.unicef.org/resources/continuity-of-essential-health-services-kenya-malawi-mozambique/	Continuity of essential health services: Study exploring the effect of Covid-19 On Demand for and Utilization of Maternal, New-born & Child Health Services	Kenya	Multi-country cross-sectional study	Service delivery, health workforce & supplies and stock, financing	MNCH & Immunization	Policy, & facility mainly and some aspects of the community	Balancing health emergency response measures with efforts to maintain regular health system function	Data extraction sheet
SR-2	Dec 2021	https://data.unicef.org/resources/continuity-of-essential-health-services-kenya-malawi-mozambique/	Continuity of essential health services: Study exploring the effect of the Covid-19 Pandemic on the Demand for, and Utilization of, Maternal, New-born and Child Health Services in Mozambique	Mozambique	Multi-country cross-sectional study	Service delivery & health workforce	MNCH	Policy, facility & community	Increase uptake of MNCH services	Data extraction sheet
SR-3	Dec 2021	https://data.unicef.org/resources/continuity-of-essential-health-services-kenya-malawi-mozambique/	Continuity of essential Health Services study exploring the effect of the Covid-19 Pandemic on the Demand for, and Utilization of, Maternal, New-born, and Child Health Services in Malawi	Malawi	Multi-country cross-sectional study	Service delivery & health workforce	MNCH	Policy & facility level	Uptake of essential MNCH, immunization, and children under 5 years of age services	Data extraction sheet
SR-4	Nov 2021	https://eurohealthobservatory.who.int/publications/i/health-systems-resilience-during-covid-19-lessons-for-building-back-better	Health systems resilience during COVID-19 Lessons for building back better	Bosnia and Herzegovina, Kyrgyzstan, Russian Federation, Ukraine, Albania, Belarus, Bulgaria, North Macedonia and Serbia)	Guidance document	Leadership and governance Health financing, health workforce, service delivery	Health system strengthening (resilience)		Support countries in building back better	Data extraction sheet
SR-5	2021	https://apps.who.int/iris/handle/10665/344925	Governance Strategies for building health system resilience	Europe (including LMICS in Europe)	Editorial	Leadership and governance	Health system strengthening (resilience)	Policy/system level	Strengthen the governance of our health systems now to ensure better resilience and performance	Data extraction sheet
SR-6	2021	https://www.who.int/publications/i/item/sea-whe-7	Learning from the COVID-19 response to strengthen health security and health systems resilience in the WHO South-East Asia Region	Southeast Asian countries (including LMICS)	Report	All 6 building blocks	Health system strengthening (resilience) and health security	Regional, policy (national), facility & community	Strengthen health security and health systems resilience in the WHO South-East Asia Region	Data extraction sheet
SR-7	2021	https://apps.who.int/iris/handle/10665/351484	Roles of community health workers in advancing health security and resilient health systems: emerging lessons from the COVID-19 response in the South-East Asia Region	Bangladesh, India, Myanmar, Thailand and Indonesia	Rapid review	Health workforce	Routine outreach services including COVID-19 response	Community level	Advancing health security and resilient health system through CHWs	Data extraction sheet
SR-8	2020	https://apps.who.int/iris/handle/10665/349644	Maintaining essential health services during COVID-19: Stories of Resilience and Innovations from 11 states of India	India	Report (stories of resilience)	All 6 building blocks	Routine services of SRMNCAH	Facility and community both	Health system resilience	Data extraction sheet
SR-9	2022	https://apps.who.int/iris/bitstream/handle/10665/363668/9789290234821-eng.pdf?sequence=3	Essential health care service disruption due to COVID-19: Lessons for Sustainability in Nigeria	Nigeria	Policy briefs	All 6 building blocks	All essential health services	Facility and community both	Sustaining essential health services	Data extraction sheet
SR-10	2022	https://apps.who.int/iris/handle/10665/365312	BUILD BACK BETTER Role of frontline health workers in managing the COVID-19 pandemic and delivery of essential health services in Assam	India	Report	Service delivery, health workforce, information system	All essential health services	Facility and community both	Sustaining essential health services (building back better)	Data extraction sheet
SR-11	2021	https://apps.who.int/iris/handle/10665/343752	COVID-19 and measures to ‘build back better’ essential health services to achieve UHC and the health-related SDGs	Southeast Asia member states including all LMICS	Report/updates (regional committee document)	Service delivery, health workforce, health financing, HMIS	Essential services	All levels (regional, national, facility and community)	Building back better	Data extraction sheet
SR-12	2021	https://www.who.int/publications/i/item/9789240040595	Maintaining the provision and use of services for maternal, new-born, child, and adolescent health and older people during the COVID-19 pandemic Lessons learned from 19 countries	Cameroon, Congo, Ethiopia, Nigeria, South Africa, Uganda, Bolivia, Brazil, Pakistan, Sudan, Tajikistan, Bangladesh India, Myanmar, Nepal and Timor-Leste	Report	All except leadership and governance	MNCAH health and ageing health services (MNCAH)	Both	Maintaining service provision	Data extraction sheet
SR-13	2021	https://apps.who.int/iris/handle/10665/338482	Impact of Covid-19 On SRMNCAH Services, Regional Strategies, Solutions, and Innovations: A Comprehensive Report: Joint WHO-UNICEF-UNFPA Initiative for SRMNCAH Programme Officers from the ministries of health and UN partners	Bhutan, India, Indonesia, Maldives, Myanmar, Sri Lanka, Thailand, Timor-Leste, Bangladesh, and DPR Korea	Report	All 6 building blocks	SRMNCAH	Both	Continuing SRMNCAH services	Data extraction sheet
SR-14	2021	https://apps.who.int/iris/handle/10665/350527	Containment strategies: lessons from early COVID-19 responses in five African countries	Kenya, Rwanda, Ethiopia, Senegal & Nigeria	Report	All 6 building blocks	COVID-19 measures predominately focus on the prevention	System & facility	Containment measures to prevent the spread	Data extraction sheet
SR-15	2021	https://www.ncbi.nlm.nih.gov/pmc/articles/PMC8451960/	Morocco’s National Response to the COVID-19 Pandemic: Public Health Challenges and Lessons Learned	Morocco	Journal article	All 6	COVID-19 measures	System & facility	N/A	Data extraction sheet
SR-16	2021	https://www.liebertpub.com/doi/epdf/10.1089/hs.2021.0094	African National Public Health Institutes Responses to COVID-19: Innovations, Systems Changes, and Challenges	South Africa, Nigeria, Uganda, Ethiopia & Mozambique)	Journal article	All 6	COVID-19 measures predominately	System, facility & community	Not mentioned	Data extraction sheet
SR-17	May 2022	https://www.ncbi.nlm.nih.gov/pmc/articles/PMC9073814/	Barriers and facilitators of access to maternal, new-born and child health services during the first wave of COVID-19 pandemic in Nigeria: findings from a qualitative study	Nigeria	Journal article	Service delivery	MNCH	Facility	Continuation of essential services such as MNCH and immunization services during COVID-19	Data extraction sheet
SR-18	May 2022	https://pubmed.ncbi.nlm.nih.gov/35291514/	Actions and Adaptations Implemented for Maternal, New-born, and Child Health Service Provision During the Early Phase of the COVID-19 Pandemic in Lagos, Nigeria: Qualitative Study of Health Facility Leaders	Nigeria	Journal article	Service delivery	MNCH	Facility	Optimizing health service provision during the early phase of the COVID-19 pandemic	Data extraction sheet
SR-19	Dec 2022	https://pubmed.ncbi.nlm.nih.gov/35867009/	Understanding changes made to reproductive, maternal, new-born and child health services in Pakistan during the COVID-19 pandemic: a qualitative study	Pakistan	Journal article	Service delivery	RMNCHN	District headquarters, rural health centres or basic health units	Prevention of delayed or forgone care during a pandemic	Data extraction sheet
SR-20	Mar, 2022	https://pubmed.ncbi.nlm.nih.gov/35321675/	Keeping essential reproductive, maternal, and child health services available during COVID-19 in Kenya, Mozambique, Uganda, and Zimbabwe: analysis of early-pandemic policy guidelines	Kenya, Mozambique, Uganda, and Zimbabwe	Policy guidelines	Service delivery, health workforce	RMNCH	Facility and outreach	To reduce potential impacts to populations related to RMNCH service delivery during COVID-19	Data extraction sheet
SR-21	Aug 2022	https://pubmed.ncbi.nlm.nih.gov/36041833/	Adapting High Impact Practices in Family Planning During the COVID-19 Pandemic: Experiences from Kenya, Nigeria, and Zimbabwe	Kenya, Nigeria, and Zimbabwe	Article	Service delivery, demand creation and enabling environment	Family planning	Facility and outreach	Adapting high impact practices during COVID-19	Data extraction sheet
SR-22	Jul-19	https://pubmed.ncbi.nlm.nih.gov/34281582/	COVID-19 in Ghana: challenges and countermeasures for maternal health service delivery in public Health facilities	Ghana	Situational update	Service delivery	MHC	Public health facilities	To mitigate the downward trend in maternal health indicators in the region. To mitigate the impact of COVID-19 restrictions	Data extraction sheet
SR-23	Jan-22	https://pubmed.ncbi.nlm.nih.gov/35012970/	Disruptions in maternal health service use during the COVID-19 pandemic in 2020: experiences from 37 health facilities in low-income and middle-income countries	Haiti, Lesotho, Liberia, Malawi, Mexico, Sierra Leone	Review	Service delivery	Maternal health	Facility	To prevent a decrease in the use of maternal health services in all countries included in the study except Malawi during the COVID-19 pandemic in 2020	Data extraction sheet
SR-24	2020	https://pubmed.ncbi.nlm.nih.gov/34521500/	Innovation in Primary health care responses to COVID-19 in Sub-Saharan Africa	Botswana, Ghana, Nigeria, South Africa, Uganda, and Zimbabwe	Qualitative descriptive study	Health workforce and service delivery	MCH, HIV, TB and non-communicable diseases (NCDs)	Facility	Innovations/actions ensure maintaining essential primary care services and preventing excess mortality from neglect of other conditions especially NCDs (diabetes, hypertension), HIV, tuberculosis, and malaria	Data extraction sheet
SR-25	Jan-23	https://apps.who.int/iris/handle/10665/351493	Maintaining essential health services during the pandemic in Bangladesh: the role of primary health care supported by routine health information system	Bangladesh	Report	Unspecified	All essential health services	Facility	Mitigate and build resilient PHC facilities in Bangladesh	Data extraction sheet
SR-26	Feb 2021	https://apps.who.int/iris/bitstream/handle/10665/351477/SEAJPH2021V10S1P26-eng.pdf?sequence=1	Emerging good practices and lessons learned to maintain essential health services during the COVID-19 Pandemic	South-East Asian Countries	Report	All 6 building blocks	All essential health services	Facility	To overcome the disruption of essential health services in the South-East Asia Region	Data extraction sheet

**Table 8:** Preferred reporting items for systematic reviews and meta-analyses extension for scoping reviews (PRISMA-ScR) checklist

Section	Item	PRISMA-ScR checklist item	Reported on page #
TITLE			
Title	1	Study Title	1
ABSTRACT			
Structured summary	2	Provide a structured summary that includes (as applicable): the background, objectives, eligibility criteria, sources of evidence, charting methods, results, and conclusions that relate to the review questions and objectives	1
INTRODUCTION			
Rationale	3	Describe the rationale for the review in the context of what is already known. Explain why the review questions/objectives lend themselves to a scoping review approach	2–3
Objectives	4	Provide an explicit statement of the questions and objectives being addressed with reference to their key elements (e.g. population or participants, concepts and context) or other relevant key elements used to conceptualize the review questions and/or objectives	3
METHODS			
Protocol and registration	5	Indicate whether a review protocol exists; state if and where it can be accessed (e.g. a Web address); and if available, provide registration information, including the registration number	3
Eligibility criteria	6	Specify characteristics of the sources of evidence used as eligibility criteria (e.g. years considered, language and publication status), and provide a rationale	5
Information sources	7	Describe all information sources in the search (e.g. databases with dates of coverage and contact with authors to identify additional sources), as well as the date the most recent search was executed	4
Search	8	Present the full electronic search strategy for at least one database, including any limits used, such that it could be repeated	4 (Table 1)
Selection of sources of evidence	9	State the process for selecting sources of evidence (i.e. screening and eligibility) included in the scoping review	3–5
Data charting process	10	Describe the methods of charting data from the included sources of evidence (e.g. calibrated forms or forms that have been tested by the team before their use, and whether data charting was done independently or in duplicate) and any processes for obtaining and confirming data from investigators	4
Data items	11	List and define all variables for which data were sought and any assumptions and simplifications made	6 (Also Table 3)
Critical appraisal of individual sources of evidence	12	If done, provide a rationale for conducting a critical appraisal of included sources of evidence; describe the methods used and how this information was used in any data synthesis (if appropriate)	N/A
Synthesis of results	13	Describe the methods of handling and summarizing the data that were charted	3–4, & 6
RESULTS			
Selection of sources of evidence	14	Give numbers of sources of evidence screened, assessed for eligibility, and included in the review, with reasons for exclusions at each stage, ideally using a flow diagram	6 Fig. 1
Characteristics of sources of evidence	15	For each source of evidence, present characteristics for which data were charted and provide the citations	45–52
Critical appraisal within sources of evidence	16	If done, present data on critical appraisal of included sources of evidence (see item 12)	N/A
Results of individual sources of evidence	17	For each included source of evidence, present the relevant data that were charted that relate to the review questions and objectives	8–36 (Table 5)
Synthesis of results	18	Summarize and/or present the charting results as they relate to the review questions and objectives	37–42
DISCUSSION			
Summary of evidence	19	Summarize the main results (including an overview of concepts, themes and types of evidence available), link to the review questions and objectives, and consider the relevance to key groups	42–43
Limitations	20	Discuss the limitations of the scoping review process	44
Conclusions	21	Provide a general interpretation of the results with respect to the review questions and objectives, as well as potential implications and/or next steps	44
FUNDING			
Funding	22	Describe sources of funding for the included sources of evidence, as well as sources of funding for the scoping review. Describe the role of the funders of the scoping review	55

## Abbreviations

AIDS: Acquired Immune Deficiency Syndrome
AKU-ERC: Aga Khan University Ethical Review Committee
ANC: Antenatal Care
CHWs: Community Health Workers
CMWs: Community Midwives
COVID-19: Coronavirus Disease 2019
EmONC: Emergency Obstetric and Newborn Care
EPI: Expanded Program on Immunisation
FP: Family Planning
GBV: Gender Based Violence
HBWs: Health Education Workers
HICs: High Income Countries
HIV: Human Immunodeficiency Virus
IMCI: Integrated Management of Childhood Illness
IMF: International Monitory Fund
IPC: Infection, Prevention and Control
IRIS: Institutional Repository for Information Sharing
JBI: Joanna Briggs Institute
LHVs: Lady Health Visitors
LHWs: Lady Health Workers
LMICs: Low and Middle Income Countries
MCH: Maternal and Child Healthcare
MeSH: Medical Subject Headings
NGO: Non-Governmental Organizations
NLM: National Library of Medicine
PHC: Primary Health Care
PMTCT: Prevention of Mother-to-Child Transmission
PNC: Postnatal Care
PPE: Personal Protective Equipment
PRISMA-ScR: Preferred Reporting Items for Systematic reviews and Meta-Analyses extension for Scoping Reviews
RMNCAH: Reproductive, Maternal, Neonatal, Child and Adolescent Health
RMNCAHN: Reproductive, Maternal, Newborn, Child, and Adolescent Health and Nutrition
SARS-CoV-2: Severe Acute Respiratory Syndrome Coronavirus 2
SOPs: Standard Operating Procedures
SRMNCH: Sexual, Reproductive, Maternal, Newborn, and Child Health Services
SRMNCAH: Sexual, Reproductive,Maternal, Newborn, Child, and Adolescent Health
TB: Tuberculosis
UHC: Universal Health Coverage
UMICs: Upper Middle-Income Countries
VHWs: Village Health Workers
WHO: World Health Organisation
