# Supplementary material for: Resilience of primary healthcare system across low- and middle-income countries during COVID-19 pandemic: a scoping review
Source: Health Res Policy Syst. 2023 Sep 18;21:98. doi: 10.1186/s12961-023-01031-4 (PMC10507852; doi:10.1186/s12961-023-01031-4)
Supplement: Supplementary file 3 — Additional file 3. ERC Letter [file 12961_2023_1031_MOESM3_ESM.pdf]

THE AGA KHAN UNIVERSITY

01-Dec-2022

Ms. NOUSHEEN PRADHAN
Department of Community Health Sciences
Aga Khan University
Karachi

Dear Ms. NOUSHEEN PRADHAN,

2022-8270-23510. NOUSHEEN PRADHAN: Resilience of Primary Health Care System Across Low- and Middle-Income Countries during COVID-19 Pandemic: A Scoping Review

Thank you for your application for exemption from ethical approval regarding the above mentioned study.

Your study was reviewed and approved as exemption. Please ensure that the study is performed as per protocol following all AKU standards.

List of document(s) submitted with this application.

Submission Document Name	Submission Document Date	Submission Document Version
Annexure 1 (1)	25-Nov-2022	2
Annexure 2 (1)	25-Nov-2022	2
nousheen - citiCompletionCertificate_7895510_48019300-Nousheen	25-Nov-2022	1
DOC-20220908-WA0003.	25-Nov-2022	
Protocol_ scoping review	30-Nov-2022	2

You may proceed with the study.

Thank you.

Sincerely,

J. Talati.

Dr. Jamsheer Talati

Chairperson
Ethics Review Committee
