# Supplementary material for: Resilience of primary healthcare system across low- and middle-income countries during COVID-19 pandemic: a scoping review
Source: Health Res Policy Syst. 2023 Sep 18;21:98. doi: 10.1186/s12961-023-01031-4 (PMC10507852; doi:10.1186/s12961-023-01031-4)
Supplement: Supplementary file 4 — Additional file 4. Corresponding references [file 12961_2023_1031_MOESM4_ESM.docx]

**Additional file 04**

**References correspond to scoping review document code.**

| **Reference as per Manuscript** | **SR document code** | **Study titles (Link)** |
| --- | --- | --- |
| 25 | SR-1 | Continuity of essential health services study Exploring the Effect Of Covid-19 On Demand For and Utilization of Maternal, New-born & Child Health Services |
| 30 | SR-2 | Continuity of essential health services study: Exploring the Effect of the Covid-19 Pandemic on the Demand for, and Utilization of, Maternal, New-born, and Child Health Services in Malawi |
| 26 | SR-3 | Continuity of essential health services study: Exploring the Effect of the Covid-19 Pandemic on the Demand for, and Utilization of, Maternal, New-born, and Child Health Services in Mozambique |
| 1 | SR-4 | Health systems resilience during COVID-19 Lessons for building back better |
| 27 | SR-5 | Editorial: governance strategies for building health system resilience |
| 32 | SR-6 | Learning from the COVID-19 response to strengthen health security and health systems resilience in the WHO South-East Asia Region |
| 37 | SR-7 | Roles of Community Health Workers in advancing health security and resilient health systems: emerging lessons from the COVID-19 Response in the South-East Asia Region |
| 38 | SR-8 | Maintaining essential health services during COVID-19: Select stories of resilience and innovations from 11 states |
| 33 | SR-9 | Essential health care service disruption due to COVID-19: Lessons for Sustainability in Nigeria |
| 42 | SR-10 | Build back better: role of frontline health workers in Managing the COVID-19 Pandemic and Delivery of essential health services in Assam |
| 39 | SR-11 | COVID-19 and measures to ‘build back better’ essential health services to achieve UHC and the health-related SDGs |
| 34 | SR-12 | Maintaining the provision and use of services for maternal, newborn, child and adolescent health and older people during the COVID-19 pandemic: lessons learned from 19 countries |
| 28 | SR-13 | Impact of Covid-19 on SRMNCAH services, regional strategies, solutions, and innovations: a comprehensive report |
| 24 | SR-14 | Containment strategies: lessons from early COVID-19 responses in five African countries |
| 31 | SR-15 | Morocco's National Response to the COVID-19 Pandemic: Public Health Challenges and Lessons Learned |
| 43 | SR-16 | African National Public Health Institutes Responses to COVID-19: Innovations, Systems Changes, and Challenges |
| 47 | SR-17 | Barriers and facilitators of access to maternal, new-born and child health services during the first wave of COVID-19 pandemic in Nigeria: findings from a qualitative study |
| 36 | SR-18 | Actions and Adaptations Implemented for Maternal, New-born, and Child Health Service Provision During the Early Phase of the COVID-19 Pandemic in Lagos, Nigeria: Qualitative Study of Health Facility Leaders |
| 48 | SR-19 | Understanding changes made to reproductive, maternal, new-born, and child health services in Pakistan during the COVID-19 pandemic: a qualitative study |
| 29 | SR-20 | Keeping essential reproductive, maternal, and child health services available during COVID-19 in Kenya, Mozambique, Uganda, and Zimbabwe: analysis of early-pandemic policy guidelines |
| 45 | SR-21 | Adapting High Impact Practices in Family Planning During the COVID-19 Pandemic: Experiences from Kenya, Nigeria, and Zimbabwe |
| 44 | SR-22 | COVID-19 in Ghana: challenges and countermeasures for maternal health service delivery in public Health facilities |
| 46 | SR-23 | Disruptions in maternal health service use during the COVID-19 pandemic in 2020: experiences from 37 health facilities in low-income and middle-income countries |
| 35 | SR-24 | Innovation in Primary health care responses to COVID-19 in Sub-Saharan Africa |
| 8 | SR-25 | Maintaining essential health services during the pandemic in Bangladesh: the role of primary health care supported by routine health information system |
| 40 | SR-26 | Emerging good practices and lessons learned to maintain essential health services during the COVID-19 Pandemic |
